# Geospatiotemporal and Causal Inferential Study of European Epidemiological Patterns of Cannabis- and Substance-Related Congenital Orofacial Anomalies

**DOI:** 10.3390/jox13010006

**Published:** 2023-02-01

**Authors:** Albert Stuart Reece, Gary Kenneth Hulse

**Affiliations:** 1 Division of Psychiatry, University of Western Australia, Crawley, WA 6009, Australia; 2 School of Medical and Health Sciences, Edith Cowan University, Joondalup, WA 6027, Australia

**Keywords:** tobacco, alcohol, cannabis, cannabinoid, cancer, cancerogenesis, mutagenesis, oncogenesis, genotoxicity, epigenotoxicity, transgenerational inheritance

## Abstract

Introduction. Since high rates of congenital anomalies (CAs), including facial CAs (FCAs), causally attributed to antenatal and community cannabis use have been reported in several recent series, it was of interest to examine this subject in detail in Europe. Methods. CA data were taken from the EUROCAT database. Drug exposure data were downloaded from the European Monitoring Centre for Drugs and Drug Addiction (EMCDDA). Income was taken from the World Bank’s online sources. Results. On the bivariate maps of both orofacial clefts and holoprosencephaly against resin, the Δ9-tetrahydrocannabinol concentration rates of both covariates increased together in France, Bulgaria, and the Netherlands. In the bivariate analysis, the anomalies could be ranked by the minimum E-value (mEV) as congenital glaucoma > congenital cataract > choanal atresia > cleft lip ± cleft palate > holoprosencephaly > orofacial clefts > ear, face, and neck anomalies. When nations with increasing daily use were compared to those without, the former had generally higher rates of FCAs (*p* = 0.0281). In the inverse probability weighted panel regression, the sequence of anomalies—orofacial clefts, anotia, congenital cataract, and holoprosencephaly—had positive and significant cannabis coefficients of *p* = 2.65 × 10^−5^, 1.04 × 10^−8^, 5.88 × 10^−16^, and 3.21 × 10^−13^, respectively. In the geospatial regression, the same series of FCAs had positive and significant regression terms for cannabis of *p* = 8.86 × 10^−9^, 0.0011, 3.36 × 10^−8^, and 0.0015, respectively. Some 25/28 (89.3%) E-value estimates and 14/28 (50%) mEVs were >9 (considered to be in the high range), and 100% of both were >1.25 (understood to be in the causal range). Conclusion. Rising cannabis use is associated with all the FCAs and fulfils the epidemiological criteria for causality. The data indicate particular concerns relating to brain development and exponential genotoxic dose-responses, urging caution with regard to community cannabinoid penetration.

## 1. Introduction

Previous reports from Hawaii, Colorado, and the USA in general [1,2,3,4] demonstrate the close link between community prenatal cannabis exposure and congenital anomalies (CAs) affecting the orofacial region (FCAs). The first study to identify FCAs in human populations was conducted in Hawaii [1]. In that study, both cleft palate (O.R. = 14.73, 95%C.I. 3.98–38.23) and cleft lip ± cleft palate (8.19, 2.22–21.13) were found to be linked to prenatal cannabis exposure. In Colorado, choanal atresia was found to be related to cannabis use [2]. In the USA, microtia/anotia, holoprosencephaly, and cleft palate alone were determined to be related to Δ9-tetrahydrocannabinol (THC) exposure [5]. For these reasons, we were keen to study these anomalies in the very important European datasets.

Orofacial congenital anomalies are some of the best-known anomalies and also some of the most obvious. Beyond this, however, they are of importance because the organizer of the face is in bidirectional communication with the organizer of the forebrain developmentally, and anomalies of the face are often associated with disorders of thinking and intellectual development [6,7]. Moreover, both are controlled overall by the gradients of the major embryonic morphogen sonic hedgehog coming from the ventral surface of the embryo, and the interruption of or interference with these gradients has been shown to lead to major defects in the development of both the face and brain [6]. This important feature increases the importance and overall relevance of the present study significantly.

It is worth noting that the eye actually develops as a composite outgrowth from both the face and the forebrain. The neural elements of the retina and optic nerve come from the forebrain outgrowth, while the more anterior parts of the eye are derived from the face. Eye anomalies as a group are considered in a companion paper, along with central nervous anomalies. Disorders of the anterior part of the eye are considered in this section.

Sonic hedgehog is a major morphogen controlling face and brain formation. It is also a major morphogen for the eyes, teeth, and nasal protuberances. It is, therefore, of great relevance to this section to note that both THC and cannabidiol have been shown to inhibit sonic hedgehog both directly [7] and via epigenetic mechanisms [8]. Other key embryological morphogens are similarly inhibited by cannabinoids, and these are discussed further in the Discussion section.

The major genotoxic cellular and molecular mechanisms relating to cannabinoids have been known for over 50 years. They include grossly abnormal sperm morphology [9,10], major loss of oocytes during cell division [11], single- and double-stranded DNA and chromosomal breaks [10,12,13,14], end-to-end chromosomal fusions [10], chromosome bridges [11,15,16,17], probable breakage-fusion-bridge cycles during testicular cancer oncogenesis [18], and abnormalities in DNA methylation [8,19,20,21,22,23,24,25,26] and both histone formation and post-translational modifications [27,28,29], which are each heritable to following generations via sperm [8,19,20,21,22,23,24,25,26,30]. A particular focus of the Discussion section of this paper will be on new and important data relating to the salience of epigenetic pathways [8]. 

The indirect mitochondrial metabolic pathways are also important for cannabinoids, as the mitochondria supply both energy and substrates, maintain a delicate mitonuclear balance, and have mitohormetic input to nuclear metabolism, which when disrupted can perturb major genomic and epigenomic energy-dependent reactions. Moreover, the mitochondria supply most of the epigenetic substrates required for epigenetic reactions. Since many cannabinoids interfere with mitochondria metabolism, this necessarily implies downstream disruption of the genomic and epigenomic homeostatic mechanisms.

One of the major features of laboratory studies of cannabinoid genotoxicity is the exponential effects of higher doses [31,32,33,34,35,36,37]. This remark applies equally to direct genotoxicity assays [7,12,31,32,33,35,36,37,38,39,40] and also to studies of the inhibition of mitochondrial metabolism [34,41,42,43,44,45], which in turn provide the organic basis of epigenomic reactions from both energy and substrate supply. Moreover, this result has been borne out in several recent epidemiological studies, which confirm this exponential effect at higher dose ranges in field studies of human populations. Since Europe, like many other places, has recently experienced a rise in cannabis use prevalence, cannabis use daily intensity, and cannabinoid THC potency, it seems that with all three trends operative in the direction of increased cannabis exposure, Europe has recently become subject to greatly increased cannabis exposure across whole populations [46,47]. This effect will be exacerbated by the long half-life of cannabinoids in the fat stores of the adipose tissue, brain, and gonads. 

Moreover, teratological considerations indicate that cannabinoids are entering the food chain in parts of France, where many acres are cropped with cannabis and calves are being born without legs, as are human babies. In fact, the odds ratio recently reported in one press release indicated a 60-fold increase in French babies born limbless [48,49,50], which actually lies within the confidence interval of the original Hawaiian report on this issue (4.45–65.63) [1]. Such reports highlight the concerns relating to the inevitable collision between rising community cannabinoid exposure and the cannabinoid genotoxic dose-response curve.

It was shown long ago that many cannabinoids (including cannabidiol, cannabinol, cannabichromene, cannabicyclol, Δ8-, Δ9- and Δ11-tetrahydrocannabinol, and their 11-hydroxymetabolites) are genotoxic and that the genotoxic activity of these compounds relates to their central biphenolic ring, which is known as olivetol [17]. The structure of olivetol is that of a central dihydroxylated benzene ring with a short aliphatic tail. Benzene is a well-established classical mutagen, teratogen, and carcinogen, whose activities have been widely recognized for many decades [51]. 

Indeed, this mutagenicity and genotoxicity also extends to many other synthetic cannabinoids. Laboratory studies have demonstrated that the synthetic cannabinoids JWH-018, JWH-133, HU210, ST5-135, 5F-AKB48, AP1NAC, CP47497, WIN55212.2, and BB-22 are similarly genotoxic [32,52,53,54]. 

The present study set out to determine the extent to which orofacial congenital anomalies may be related to exposure to cannabis and other substances in the social environment at the national level across Europe. The analysis plan was to employ both bivariate and multivariable techniques to examine these relationships, and to do so within formal quantitative causal inferential and geospatiotemporal frameworks. The advent of the massive EUROCAT congenital anomaly database [55], along with recent epidemiological explorations of the European experience of cannabis exposure [56], greatly facilitated this endeavor.

## 2. Methods 

### 2.1. Data

Data on all the available congenital anomaly rates were downloaded for each individual year for the 14 nations from the European Network of Population-Based Registries for the Epidemiological Surveillance of Congenital Anomalies (EUROCAT) website [55] and then analyzed. The total congenital anomaly rate from the EUROCAT includes the anomaly rates for live births, stillbirths, and cases where early termination due to an anomaly was employed all combined together, meaning that it represents a total overall rate across all classes of births. The nations selected were chosen based on the availability of their congenital anomaly data for most of the years 2010–2019. Data on tobacco (as the percent of daily tobacco use prevalence) and alcohol (as the liters of pure alcohol consumed per capita annually) use for each country were sourced from the World Health Organization [57]. Data on drug use for cannabis, amphetamines, and cocaine was derived from the European Monitoring Centre for Drugs and Drug Addiction (EMCDDA) online database [58]. The last-month cannabis use data were also supplemented by information on the THC content of cannabis herb and resin samples published in recent reports [47], which itself was originally derived from the EMCDDA [47]. The median household income data (in $USD) were taken from online World Bank sources [59]. 

### 2.2. National Assignment 

The nations were categorized into two groups as being either high and rising daily cannabis use or low and/or falling daily cannabis use based on the categorization in a recent detailed European epidemiological study (see Figure S4 in [47]). In this way, Belgium, Croatia, the Netherlands, France, Germany, Italy, Norway, Portugal, and Spain were categorized as nations experiencing increasing daily use, while Hungary, Bulgaria, Finland, Poland, and Sweden were categorized as nations experiencing low or falling levels of daily cannabis use.

### 2.3. Derived Data

As several metrics of cannabis exposure were available, it was possible to calculate various derived metrics. Thus, the last-month cannabis use prevalence data were multiplied by the THC content of cannabis herb and resin samples to derive a compound metric from their product. These metrics were then multiplied by the imputed daily cannabis use prevalence rates to derive comprehensive additional compound metrics for both cannabis resin and herb.

### 2.4. Data Imputation 

Linear interpolation was used to complete any missing datasets. This was particularly relevant for the daily cannabis use data. A total of 59 data points on daily cannabis use were directly available from the EMCDDA for the 14 nations over the study period. The use of linear interpolation allowed for the expansion of this dataset to 129 data points (further details and documentation are provided in the Results section). Data concerning the THC concentration of cannabis resin were unavailable for Sweden. It was, however, noted that the resin-to-herb THC concentration was almost constant at 17.7 in each year in nearby Norway. This ratio was, therefore, applied to the Swedish cannabis herb THC concentration data to derive estimates of the Swedish cannabis resin THC concentration. Similarly, data concerning the cannabis resin THC concentration in Poland were not available. The annual ratio of the THC concentration of cannabis resin to the THC concentration of cannabis herb in Germany was, therefore, used to estimate the resin THC content in Poland from the known herb THC concentrations in Poland. As geospatial analytical techniques do not allow for missing data, the dataset was completed by the last observation carried forward or backwards for Croatia in 2018 and 2019 and the Netherlands in 2010. It was not appropriate to use multiple imputation methods for this dataset, as multiple datasets cannot be used as simultaneous chained inputs for panel or spatial multivariable regression techniques, and moreover, published model pooling techniques are not available for these models. 

### 2.5. Statistics 

The gathered data were processed in R Studio version 1.4.1717 based on R version 4.1.1, which was obtained from the Comprehensive R Archive Network and the R Foundation for Statistical Computing [60]. The analysis was conducted in December 2021. The data were manipulated and rearranged using dplyr from the tidyverse from the R Core development team [61]. The data were log transformed as appropriate to improve the compliance with the normality assumptions based on the results of the Shapiro–Wilk test. Two-dimensional graphs were drawn using ggplot2 from the tidyverse. Maps were made using ggplot2 and sf (simple features) [62]. Custom color palettes and palettes taken from the viridisLite and viridis packages were used for the color fills [63]. The R package colorplaner was used to compose the bivariate maps [64]. None of the illustrations have been previously published and all are original. The linear regression was conducted in Base R. The mixed effects regression was performed using the R package nlme [65]. The classical technique of the serial deletion of the least significant term was used for the reduction of all the multivariable models to yield a final reduced model, which is the model presented in the following tables. Multiple linear models were processed simultaneously using the combined coordinated techniques from the R packages purrr and broom, as is well described [61,66,67]. In multivariate interactive models, the effect of any particular covariate may not be immediately apparent. The effect of an individual covariate can be calculated from such models and is known as the marginal effect. In this study, this was calculated in R using the package margins [68].

### 2.6. Covariate Selection 

The presence of multiple different metrics for cannabis exposure created a problem in terms of the analysis, as it was unclear which was the most appropriate combination of metrics to employ for a particular multivariable model. The indiscriminate use of excessive covariates would unnecessarily consume degrees of freedom and complicate the analysis, thereby restricting the ability to assess important effects. This issue was addressed directly through the use of a random forest regression conducted using the R package ranger [69], and the variable importance was formally assessed at the same time using the R package vip (variable importance plot) [70]. The most highly predictive covariates from this process were then entered into the regression modelling equations. The tables from this analysis are presented in the Results section. 

### 2.7. Panel and Geospatial Analysis 

The panel analysis was conducted using the R package plm [71] across both space and time simultaneously utilizing the “twoways” effect. The spatial weights matrix was calculated using the edge and corner “queen” relationships (by analogy with chess) utilizing the R package spdep (spatial dependency) [72]. The geospatial modelling was conducted using the spatial panel random effects maximum likelihood (spreml) function from the R package spml, which is an advanced function that allows detailed correction of highly anomalous spatial model error structures [73,74]. Such models may produce up to four model error coefficients, which are useful in determining the optimal error structure of the model. These coefficients are phi (the random error effect), rho (the spatial coefficient), psi (the serial correlation effect), and theta (the spatial autocorrelation coefficient). The most appropriate error structure was chosen for each spatial model, taking care to preserve the model error specification between closely related models. The backwards methods obtained via reduction from the full general model to the most specific model were used to determine the most appropriate error structure, as has been previously descried [75]. Both the panel and geospatial models were temporally lagged, as indicated in the Results section, by one to two years.

### 2.8. Causal Inference 

The tools of formal causal inference were deployed in this analysis. Inverse probability weighting (ipw) is the technique of choice and converts an observational study into a pseudo-randomized study from which it has been convincingly shown to be appropriate to draw causal inferences [76]. All the multivariate panel models presented herein were inverse probability weighted. The inverse probability weighting was conducted using the R package ipw [76]. Similarly, E-values (expected values) provide a quantitative estimate of the correlation required for some hypothetical unmeasured confounder covariates with both the exposure of concern and the outcome of interest in order to explain some apparently causal relationships [77,78,79]. It is thus a very useful tool for use in a sensitivity analysis and provides a quantitative measure of the robustness of the model to extraneous covariates that have not been included within the matrix of the chosen independent covariates. E-values also have a confidence interval, and the 95% lower bound of this confidence interval is of particular importance and is widely reported in this study. E-value estimates greater than 1.25 are usually interpreted as implying causality [80], and E-values greater than nine are described in the literature as being high [81]. The R package EValue was used to calculate the E-values [82]. Both the E-values and inverse probability weighting are foundational and pivotal techniques used in quantitative formal causal inferential methods, allow causal relationships to be assessed from real-world observational studies, and essentially and powerfully transform the analytical paradigm.

### 2.9. Data Availability 

The raw datasets, including 3800 lines of computation code in R, have been made freely available through the Mendeley data repository. They can be located at the following URLs: https://doi.org/10.17632/tysn37t426.1 and https://doi.org/10.17632/jjhpfxz5m7.1.

### 2.10. Ethics 

Ethical approval for this study was provided by the Human Research Ethics Committee of the University of Western Australia (number RA/4/20/4724) on 24 September 2021.

## 3. Results

Table S1 sets out the basic background data for the study population. The table lists the 14 nations contributing data to the study and the 9 CAs in this group. It also provides drug use data, including summary statistics on the compound metrics of cannabis exposure, in addition to median household income data. 

During embryology, the eye forms as a confluence of tissues from both the face and the developing forebrain. The facial tissues contribute the tissues at the front of the eye, and the retina and neural components grow out as protuberances from the forebrain. Hence, disorders of the anterior eye are included in this section, while disorders of the eye as a whole and the posterior eye tissues are addressed in a companion manuscript, which addresses disorders of the central nervous system.

Table S2 sets out the daily cannabis use data, which was notably incomplete. A total of 59 data points were identified in this group of nations. The data were completed by means of linear interpolation, as indicated in Table S3, with the addition of a further 70 points to make 129 points for this covariate overall.

**Figure 1:**
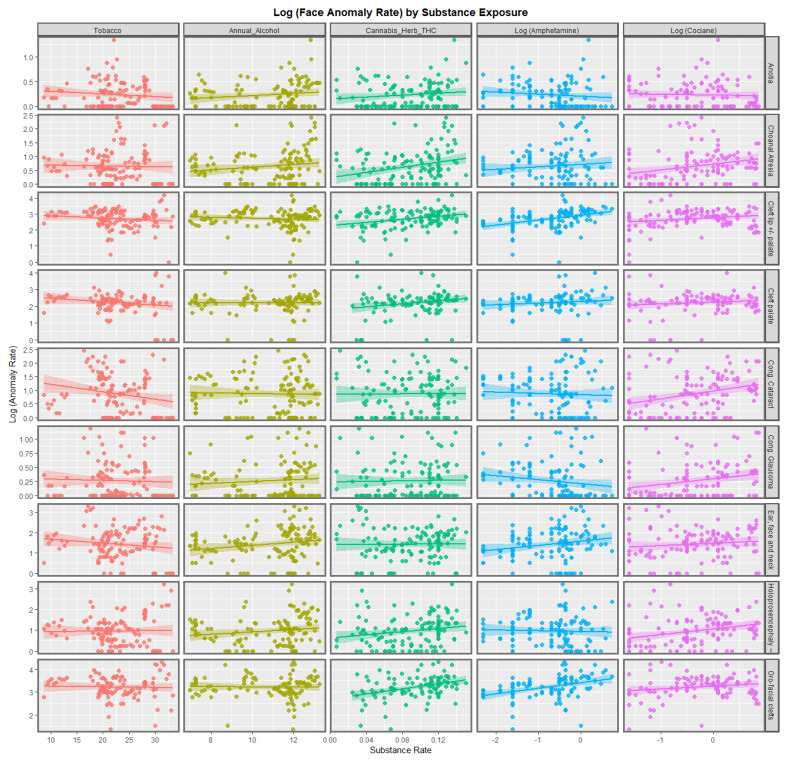
Log Rates of Orofacial Anomalies by Substance Exposure. Caption: Paneled scatterplot of the log (congenital anomaly rates) by substance exposure.

Figure 1 shows the regression lines for each of the anomalies with tobacco, alcohol, cannabis herb THC concentration, amphetamine, and cocaine. The trend lines for tobacco are either flat or have a negative slope. The trend lines for alcohol are mostly flat, although two are weakly positive. The trend lines for amphetamine exposure are either flat or have slopes in both the positive and negative directions. For the cannabis herb THC concentration, while some regression lines are flat, those for anotia, choanal atresia, cleft lip and/or palate, holoprosencephaly, and orofacial clefts appear to be rising significantly.

**Figure 2:**
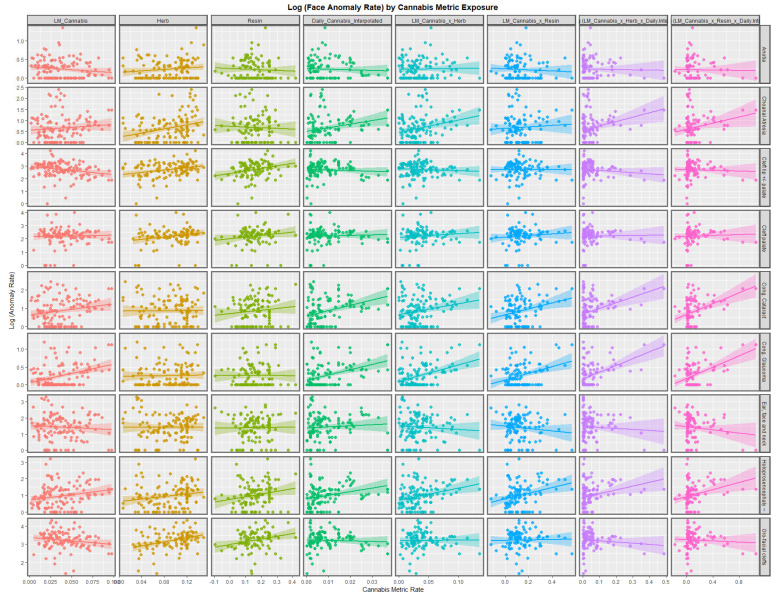
Log Rates of Orofacial Anomalies by Cannabis Exposure. Caption: Paneled scatterplot of the log (congenital anomaly rates) by exposure to various cannabis metrics.

Figure 2 illustrates the lines of best fit for the various metrics of cannabis exposure. The regression lines for the cannabis resin THC concentration and orofacial clefts and holoprosencephaly appear to be strongly positive. The daily cannabis use interpolated appears to be strongly associated with choanal atresia, congenital cataract, congenital glaucoma, and holoprosencephaly. The trend lines for the compound cannabis metrics are as indicated.

**Figure 3:**
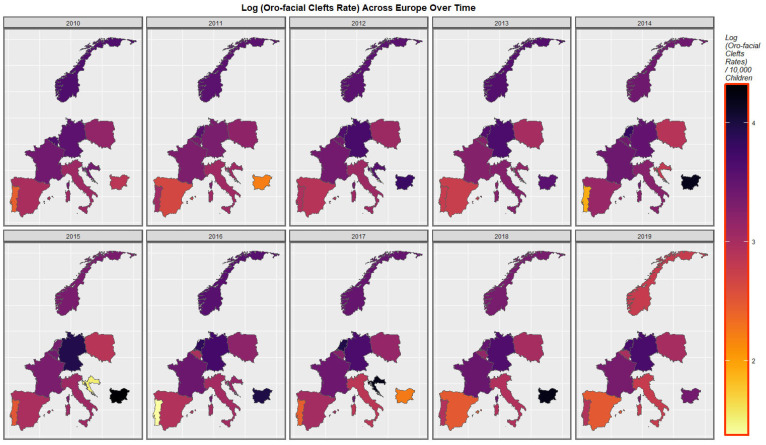
Log Orofacial Cleft Rate Over Time. Caption: Sequential map-graphs of the log (orofacial cleft rates) across the surveyed European nations over time 2010–2019.

Figure 3 is a graphical map showing the distribution over time of the orofacial clefts rates across Europe. High rates are noted at different times in the Netherlands, Germany, Croatia, and Bulgaria. 

**Figure 4:**
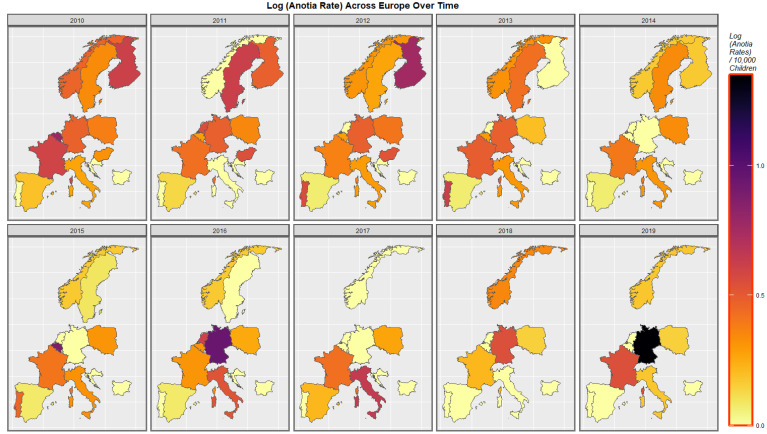
Log Anotia Rate Over Time. Caption: Sequential map-graphs of the log (anotia/microtia rates) across the surveyed European nations over time 2010–2019.

Figure 4 shows the time distribution of the anotia case rate. The French rate is noted to have waxed and waned and then risen again in 2018. The German rate is noted to have risen overall. Higher rates emerged in Belgium, the Netherlands, and Portugal at different periods.

**Figure 5:**
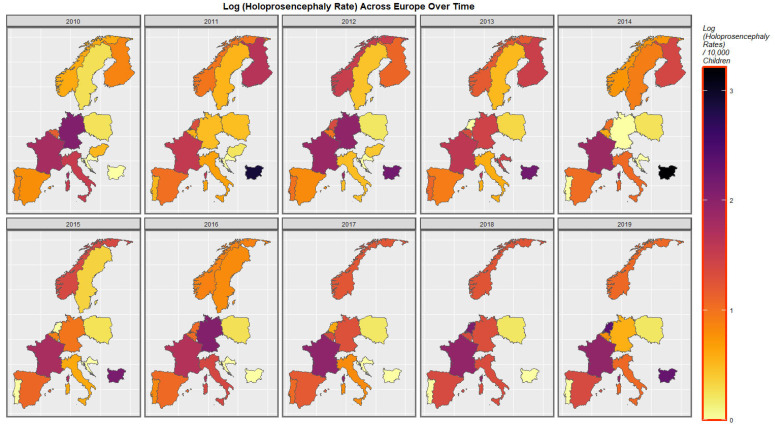
Log Holoprosencephaly Rate Over Time. Caption: Sequential map-graphs of the log (holoprosencephaly rates) across the surveyed European nations over time 2010–2019.

The time-dependent rates of holoprosencephaly and choanal atresia are similarly displayed in Figure 5 and Figure S1.

Figure S2 shows the rate of the compound cannabis exposure metric last-month cannabis use x cannabis resin THC concentration. Temporal rises in Spain, France, Italy, the Netherlands, Belgium, and Bulgaria are noted.

**Figure 6:**
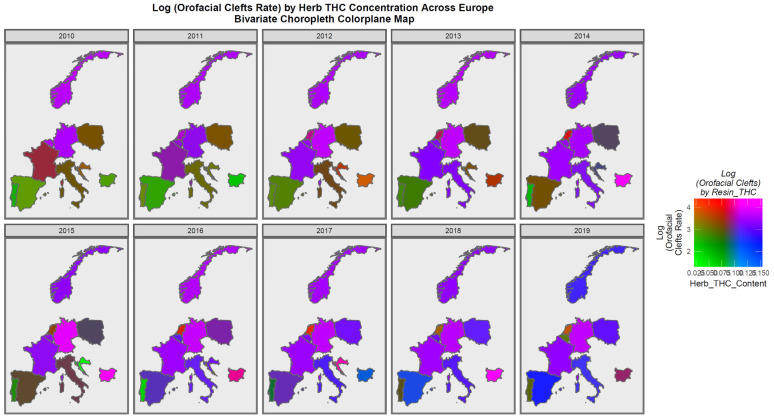
Bivariate Map of Log Orofacial Clefts by Cannabis Herb THC Concentration. Caption: Colorplaner bivariate sequential map-graphs of the log (orofacial congenital anomaly rates) by cannabis herb THC concentrations across the surveyed European nations over time 2010–2019.

Figure 6 is a bivariate plot of the relationship between orofacial clefts and the cannabis herb THC concentration. In this plot, the green shading represents areas where both covariates are low, while the pink or purple shading shows areas where both are high, as shown in the colorplane in the key. France, the Netherlands, Belgium, Norway, and Bulgaria are noted to be pink or purple at times.

**Figure 7:**
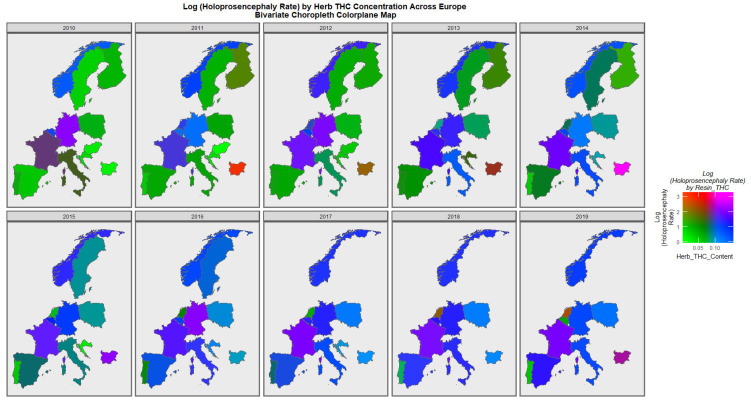
Bivariate Map of Log Holoprosencephaly by Cannabis Herb THC Concentration. Caption: Colorplaner bivariate sequential map-graphs of the log (holoprosencephaly rates) by cannabis herb THC concentrations across the surveyed European nations over time 2010–2019.

Figure 7 is a similar bivariate graphical map of the holoprosencephaly against cannabis herb THC concentration. Germany, France, and Bulgaria are noted to be pink or purple at different times.

**Figure 8:**
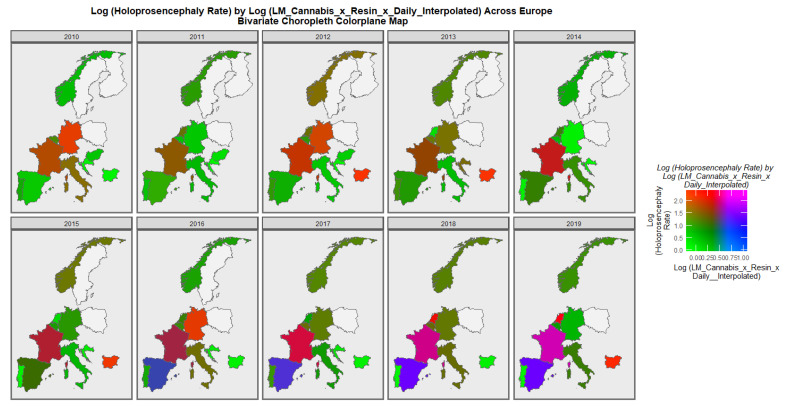
Bivariate Map of Log Holoprosencephaly by Cannabis Metric. Caption: Colorplaner bivariate sequential map-graphs of the log (holoprosencephaly rates) by log (last month cannabis use x cannabis resin THC concentration x daily use interpolated) across the surveyed European nations over time 2010–2019.

When the holoprosencephaly rate is charted against the compound metric of last-month cannabis use x resin THC concentration x daily use interpolated, the appearances shown in Figure 8 are generated. It is clear from this figure that the area of France emerges as pink, signifying that both rates are elevated.

**Figure 9:**
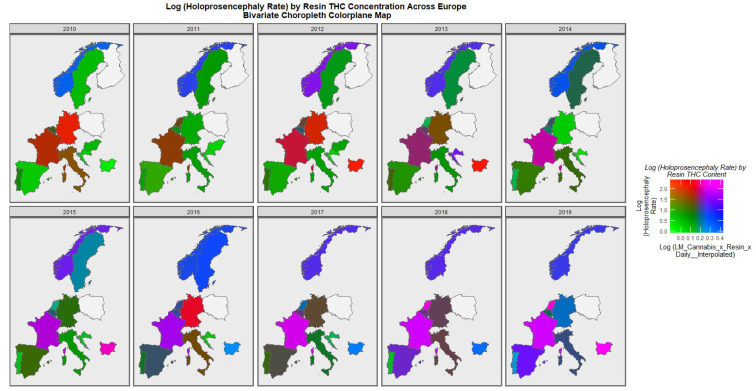
Bivariate Map of Log Holoprosencephaly by Cannabis Resin THC Concentration. Caption: Colorplaner bivariate sequential map-graphs of the log (holoprosencephaly rates) by cannabis resin THC concentrations across the surveyed European nations over time 2010–2019.

**Figure 10:**
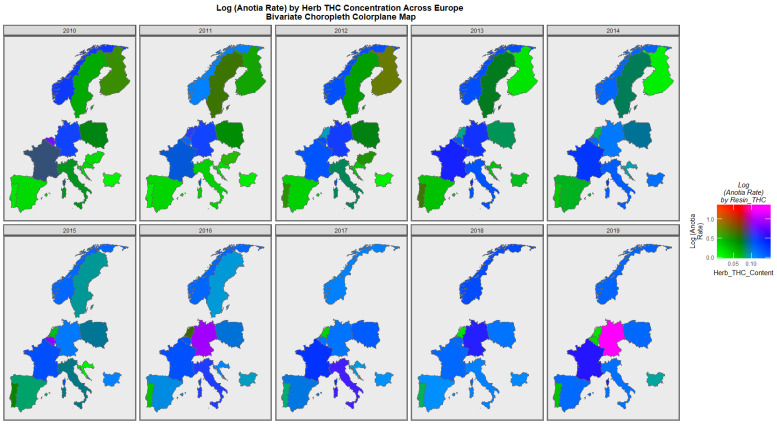
Bivariate Map of Log Anotia by Cannabis Herb THC Concentration. Caption: Colorplaner bivariate sequential map-graphs of the log (anotia/microtia rates) by cannabis herb THC concentrations across the surveyed European nations over time 2010–2019.

When the holoprosencephaly rate is charted against the cannabis resin THC concentration, France, the Netherlands, and Bulgaria are noted to turn pink or purple across the decade (Figure 9). When anotia is charted against the cannabis herb THC concentration, Germany and Belgium are noted to turn purple at times (Figure 10). When choanal atresia is charted against the cannabis herb THC concentration (Figure S3), Germany and Bulgaria are noted to be pink or purple at times.

**Figure 11:**
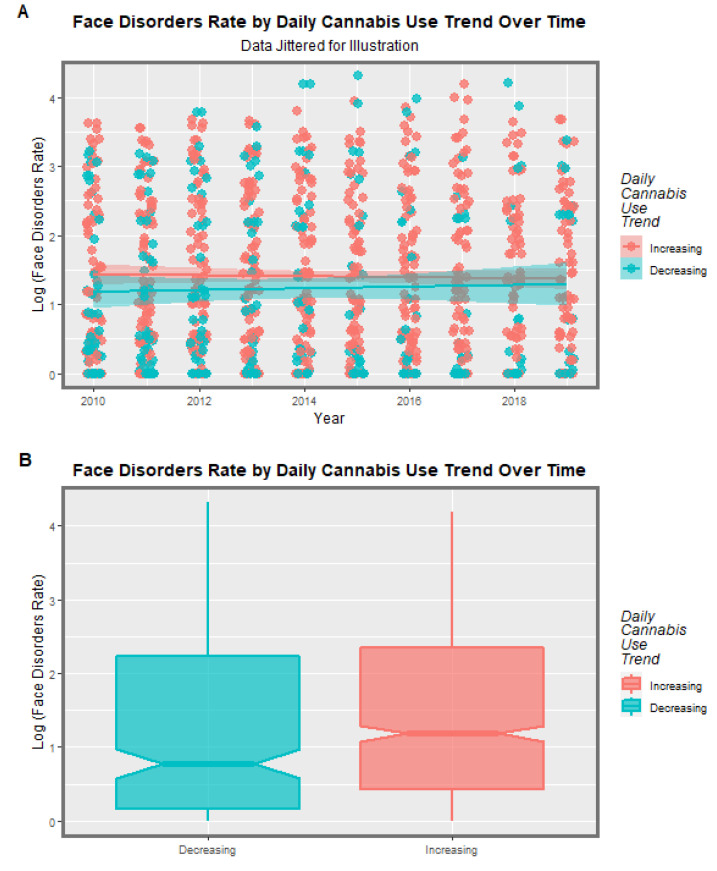
Collected Facial Disorders Aggregated Across Time. Caption: Facial congenital anomaly rates by daily cannabis use interpolated rates: (**A**) scatterplot over time and (**B**) boxplot aggregated across time. Non-overlapping notches indicate statistically significant differences.

**Figure 12:**
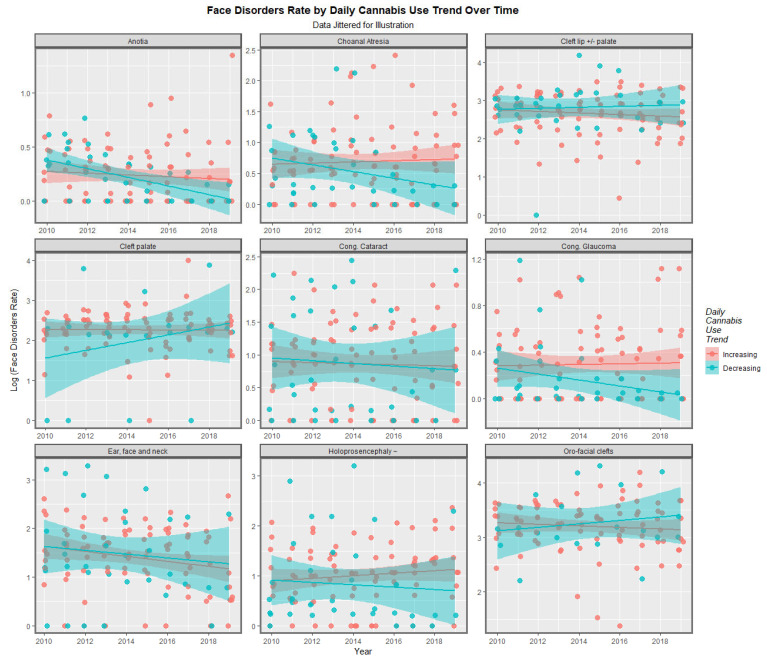
Time Trends of Individual Facial Disorders. Caption: Paneled scatterplots of the log (orofacial congenital anomaly rates) by daily cannabis use interpolated rates by anomaly.

A particular concern relates to high-intensity daily use [8,18,46,83,84,85]. For this purpose, it is of interest to divide the nations into those where daily cannabis use is high or increasing and those where it is low or declining. As described in the Methods section, this was done based on recent published reports (see eFigure 4 in [47]). When this was done, the results charted in Figure 11 were found. In the upper scatterplot, across time the nations with increasing cannabis daily use appear to be a little higher than the others. This is made explicit in the lower boxplot graph, which aggregates the data over time, where the notches on the two box plots obviously do not overlap and thus indicate a statistically significant difference in the rates (linear regression: β-est. = 0.1768, t = 2.2, *p* = 0.0281; model F = 4.83, dF = 1, 1066, *p* = 0.0281; t-test: t = 2.12, df = 4741.6, *p* = 0.0346). However, when the data are separated by the anomaly type, no apparent difference in the two groups of nations is apparent (Figure 12). 

Table S4 lists the slopes of the various bivariate regression lines shown in Figure 1 and Figure 2. The 39 slopes that are positive and statistically significant are extracted and shown in Table 1. They are listed in terms of the descending minimum E-values (mEV). A total of 33 of the listed terms include metrics of cannabis exposure, 5 relate to cocaine, and 1 to alcohol consumption. The E-value quantifies the strength of the association with both the exposure of interest and the outcome of concern to obviate some apparently causal effect. The E-value is a key metric in causal inference.

From this table, the FCAs may be ranked in order by their median mEV as congenital glaucoma (median mEV = 95.71) > congenital cataract (6.09) > choanal atresia (5.53) > holoprosencephaly and arhinencephaly (3.85) > orofacial clefts (3.26) > cleft lip ± cleft palate (2.00) > ear, face, and neck anomalies (1.07). Anotia and cleft palate alone do not feature in this list.

**Figure 13:**
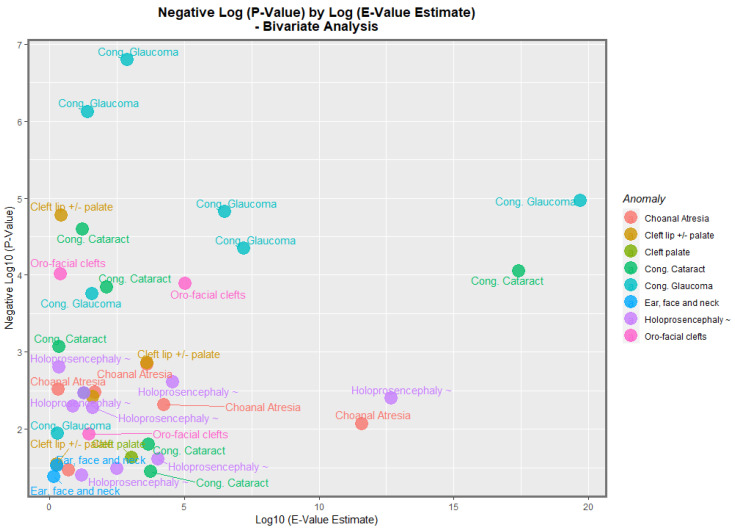
Volcano Plot, Negative Log *p*-Values Against Log E-Value Estimates. Caption: Volcano plot of the negative log of the *p*-values against the negative log of the E-value estimates, bivariate linear relationships.

By analogy with the genomic literature in which volcano plots are used to chart the negative log of the *p*-value against the fold-change in the gene expression, it is possible to chart the negative log *p*-value of these bivariate regression coefficients against the fold-change quantified as the E-value. Figure 13 does this for the negative log of the *p*-value against the E-value estimate. In this figure, congenital glaucoma, congenital cataract, and orofacial clefts figure highly in the plot.

**Figure 14:**
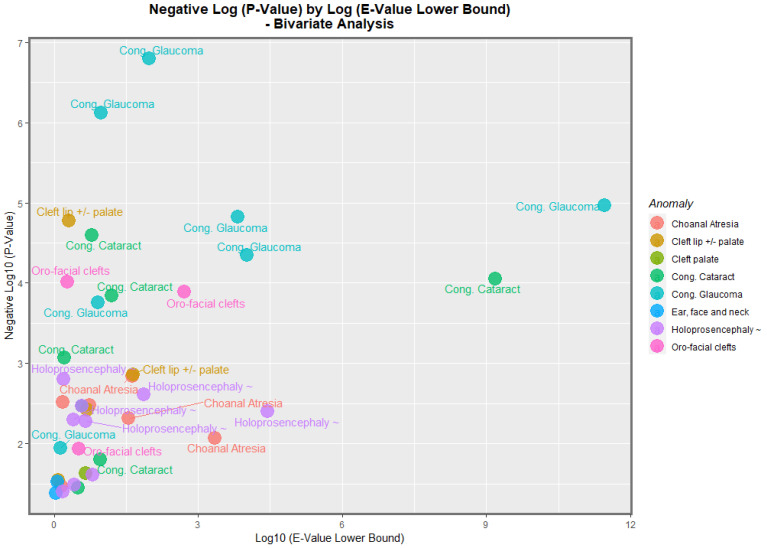
Volcano Plot, Negative Log *p*-Values Against Log 95% Minimum E-Value C.I. bounds. Caption: Volcano plot of the negative log of the *p*-values against the negative log of the minimum E-values, bivariate linear relationships.

Figure 14 makes a similar plot for the minimum E-value. The same anomalies are most prominent.

Having made these important bivariate observations, the next issue is the nature of the results considered in the multivariable regression. However, given the large number of cannabis exposure metrics, the variable selection for these regression equations is far from straightforward.

For these reasons, a random forest regression conducted in the R package ranger was used together with the variable importance package to create tables listing the relative importance of the 13 possible covariates of interest. Four variable importance tables are listed in Tables S5–S8 for the four orofacial congenital anomalies of particular interest. These were orofacial clefts, anotia/microtia, congenital cataracts, and holoprosencephaly. The reasons for choosing these particular CAs are explained in the Discussion section.

Table S9 lists the final models from the four multivariable panel regression equations for orofacial clefts shown for one each of an additive and interactive model and models lagged by one and two years. All of these models are inverse probability weighted, which transfers them from a purely observational context into a pseudo-randomized context from which one is able to meaningfully draw causal inferences. Inverse probability weighting is the technique of choice for such data transformations within the discipline of casual inference. It is noted that in each model, terms such as cannabis exposure metrics survive the model reduction, have positive regression coefficients, and are statistically significant.

Tables S10–S12 list similar models for anotia/microtia, congenital cataract, and holoprosencephaly. Similar observations can be made in each of these cases. One notes that in all the models presented in this section, terms such as metrics of cannabis exposure persist after the model reduction, are positive, and are statistically (often highly) significant.

The next issue relates to the question of what would happen if these data were considered in their formal space–time relationships. It is possible to do this formally in a way that accounts for issues which are potentially serious in data analysis, such as serial correlation, random effects, spatial error correlation, and spatial autocorrelation in the patterns of data expression.

Figure S1 shows the initial, edited, and final geospatial links between the nations, which became the basis from which the sparse spatial weights matrix was derived.

Table 2 shows the final geospatial regression models for the additive, interactive, and lagged models for orofacial clefts. One notes from this table that, once again, terms such as cannabis survive the model reduction, are positive, and are statistically highly significant. The same pattern is repeated in Table 3, Table 4 and Table 5 for anotia/microtia, congenital cataract, and holoprosencephaly.

These various regression parameters can each be associated with the E-values. The E-values applicable to the panel and geospatial regression models are listed in Table 6 and Table 7, respectively. These tables may be combined and re-presented as a complete single list, as shown in Table 8. Table 9 presents the list of 28 E-value estimates and mEVs in descending order of mEV. In this table, the link between each E-value estimate and mEV has been broken so that both lists appear in straight descending order. From Table 9, it is apparent that 25/28 (89.3%) E-value estimates are greater than 9 and so fall into the high response zone [81], and that all 28 (100%) are greater than the 1.25 cut-off for causality [80]. In terms of the mEVs, it is noted that 14/28 (50%) are greater than 9, while all 28 (100%) are greater than the 1.25 threshold indicative of a potentially causal relationship.

Table 8 is then re-presented according to the order of congenital anomalies in Table 10. Table 10 is then summarized for comparative purposes as Table 11, which lists various summary statistics for the E-values in descending order of the median mEV, which coincides with the order of the median E-value estimate. Hence, the order of association seen in this table is anotia > congenital cataract > orofacial clefts > holoprosencephaly.

**Figure 15:**
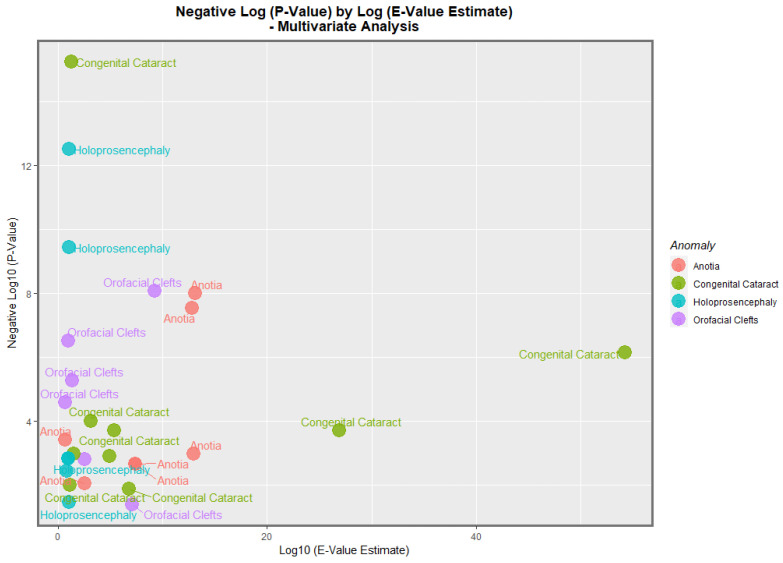
Multivariate Analysis of *p*-Values by E-Value Estimate. Caption: Volcano plot of the negative log of the *p*-values against the negative log of the minimum E-values, multivariate relationships.

**Figure 16:**
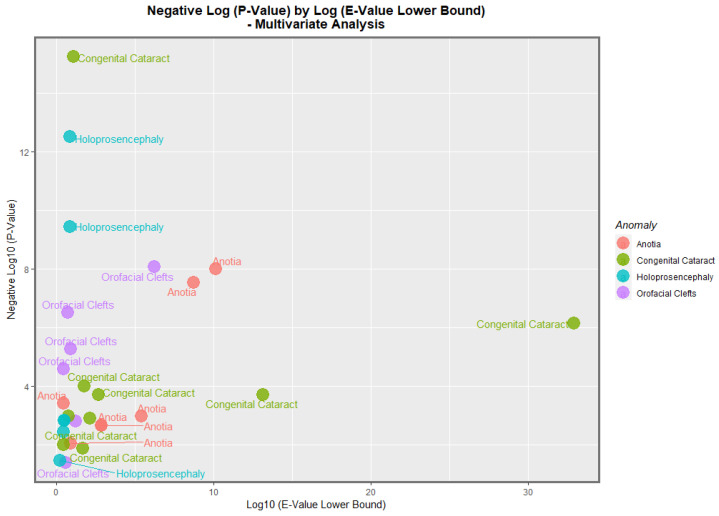
Multivariate Analysis of *p*-Values by E-Value Lower Bound. Caption: Volcano plot of the negative log of the *p*-values against the negative log of the minimum E-values, multivariate relationships.

The data from Table 8 is presented graphically in Figure 15. Here, it is noted that congenital cataract, holoprosencephaly, anotia/microtia, and orofacial clefts are all high scoring, with points seen at the periphery of the data cloud. Figure 16 presents a similar plot for the minimum E-values, with qualitatively similar results.

Table S13 re-lists the data from Table 8 ordered by the regression terms. As indicated, the regression terms can be simplified and grouped by their primary covariate into daily cannabis use interpolated, herb THC concentration, or resin THC concentration. The descriptive statistics from these data can then be summarized and ordered by descending mEV, as indicated in Table 12, where it is seen that when one considers the median E-value estimate and mEV, the order of potency is daily use interpolated > cannabis herb THC concentration > cannabis resin THC concentration.

These terms may be quantitatively compared using the Wilcoxon test, as shown in Table 12. As shown in this table, only the comparison between the daily use and the THC concentration of cannabis resin for the E-value estimate is significant (W = 36, *p* = 0.0115).

## 4. Discussion

### 4.1. Main Results

The main result of the present analysis was that seven of the nine anomalies in this group could be significantly related based on the bivariate analysis with the metrics of cannabis exposure and that all four of the FCAs chosen for further multivariable analysis could be related to the metrics of cannabis exposure on inverse probability weighted panel and geospatial modelling. Thus, overall, eight of nine FCAs were relatable to cannabis exposure, with the sole exception being the group ear, face, and neck anomalies.

### 4.2. Detailed Review of Results

According to the bivariate analysis, tobacco was not obviously related to any of the FCAs. Alcohol was related to choanal atresia; ear, face, and neck anomalies; and HPE. The cannabis herb THC concentration was related to choanal atresia and orofacial clefts, holoprosencephaly, and cleft lip ± palate. The cannabis resin THC concentration was related to cleft lip ± palate, choanal atresia, holoprosencephaly, and orofacial clefts.

The maps of the FCA incidence indicated that the anotia rates deteriorated in France and Germany; holoprosencephaly deteriorated in Spain, France, German, Bulgaria, and Norway; and choanal atresia became worse in German, France, Spain, Italy, and Hungary. The bivariate maps of orofacial clefts against the cannabis resin THC concentration showed that both covariates increased together in Norway, France, the Netherlands, and Bulgaria. When holoprosencephaly was mapped against the cannabis resin THC concentration, both covariates increased together in the Netherlands, France, and Bulgaria. When holoprosencephaly was mapped against the cannabis herb THC concentration, both covariates increased simultaneously in France, Germany, and Bulgaria. When anotia was mapped against the cannabis herb THC concentration, both covariates increased together in Belgium, the Netherlands, and Germany. When choanal atresia was mapped against the cannabis herb THC concentration, both covariates increased together in Belgium, Spain, and Germany.

When the nations with increasing daily use were compared to those without, the former group had generally higher rates of FCAs (*p* = 0.0281, Figure 11). The daily cannabis interpolated was the most significant covariate in the bivariate regression (Table 1). The most powerfully related FCAs in the bivariate volcano plots were congenital glaucoma, congenital cataract, and orofacial clefts (Figure 13 and Figure 14). From Table 1, the FCAs may be ranked in order by their median mEV as congenital glaucoma (median mEV = 95.71) > congenital cataract (6.09) > choanal atresia (5.53) > cleft lip ± cleft palate (4.58) > holoprosencephaly and arhinencephaly (3.85) > orofacial clefts (3.26) > ear, face, and neck anomalies (1.07). Anotia and cleft palate alone do not feature in this list.

Four anomalies were selected for detailed multivariable study. According to the inverse probability weighted multivariable panel regression for the sequence of anomalies, orofacial clefts, anotia, congenital cataract, and holoprosencephaly had positive and significant cannabis coefficients ranging from *p* = 2.65 × 10^−5^, 1.04 × 10^−8^, 5.88 × 10^−16^ and 3.21 × 10^−13^. According to the geospatial multivariable regression, this same series of FCAs had terms for cannabis that were positive and significant from *p* = 8.86 × 10^−9^, 00011, 3.36 × 10^−8^ and 0.0015. The most powerfully related on the multivariable volcano plots were congenital glaucoma, congenital cataract, and orofacial clefts (Figure 15 and Figure 16).

Some 25/28 (89.3%) E-value estimates and 14/28 (50%) minimum E-values exceeded 9 and thus fell in the high range [81]. Moreover, 100% of the E-value estimates and mEVs fell above the 1.25 cut-off for causal relationships [80]. Considering the median mEVs, the strength of the relationship with cannabis was anotia > congenital cataract > orofacial clefts > holoprosencephaly. Considering the mEV, the explanatory power of the cannabis metrics daily cannabis use > herb THC concentration > resin THC concentration, although these differences were (mostly) not significant.

### 4.3. Choice of Anomalies

Some explanation why the various anomalies chosen for further multivariable analysis were selected is of interest. Cleft lip ± cleft palate was chosen because it is the best-known and amongst the commonest anomalies in this group, and it is often a highly visible anomaly. Anotia was chosen because this FCA has been found to be associated with cannabis exposure in Hawaii, Colorado, the USA, and Australia [1,2,3,5]. It was, therefore, naturally of interest to see how it would perform in the European dataset. Congenital cataract was chosen because it showed large bivariate effects (Figure 1 and Figure 2). Holoprosencephaly was chosen because it showed large bivariate effects, and as it has recently been shown to be due to sonic hedgehog inhibition interfering with the face and forebrain morphogenesis, it was of prognostic interest not only for facial but also for neurological development [6,7].

### 4.4. Qualitative Causal Inference

In 1965, the great English epidemiologist A.B. Hill set out what have become the accepted qualitative criteria for demonstrating causality in an associative relationship [86]. His nine criteria were strength of association, consistency amongst studies, specificity, temporality, coherence with known data, biological plausibility, dose response curve, analogy with similar situations elsewhere, and experimental confirmation. It is clear from the above remarks that the present results fulfill all of these criteria.

### 4.5. Quantitative Causal Inference

One of the major issues faced by observational studies is the non-comparability of experimental groups. The analytical technique of choice with which to address this is inverse probability weighting. This technique has been applied to all the panel models shown in this report. In interpreting our results, it is important to appreciate that this technique moves this report from the merely associational into a pseudo-randomized paradigm from which is it entirely appropriate to make truly causal inferences.

Another issue faced by observational studies is that some uncontrolled confounding variable may exist that explain away the reported apparently causal relationships. The use of the E-value (or “Expected value”) quantifies the degree of association demanded of such a hypothetical extraneous confounder covariate with both the exposure of concern and the outcome of interest in order to obviate the results reported. Since 50% of our minimum E-values were in the high range and 71.4% were in the moderate range (greater than 5), we are confident that this is not a major issue for the results in our study.

For these reasons, we feel convinced that the present results also fulfill the criteria for quantitative formal causal inference, further lending weight to the study findings.

### 4.6. Mechanisms

#### 4.6.1. Morphogen Gradients

The centrality of the major embryonic morphogen gradients to the normal patterning and organogenic growth of embryonic development was alluded to in the Introduction. In addition to their effect on sonic hedgehog, cannabinoids have also been reported to interfere with fibroblast growth factor [87,88], retinoic acid [89,90,91], and bone morphogenetic proteins [92,93,94], amongst others. Together, such disruptions place inordinate stress on the normal embryogenic pathways, sequences, and patterning.

#### 4.6.2. Epigenomic Impacts on the Genes of Facial Developmental

##### Cannabinoid-Induced Epigenomic Perturbations

There is increasing recent concern at the epigenetic impacts and, therefore, transgenerational effects of many cannabinoids [54,95,96]. Recent serial whole epigenome screening has identified many sets of functional annotations identified in the Ingenuity Pathway Analysis of human sperm, including particularly the following, which are of relevance to the morphogenesis of facial structures [8]:

Head development (47 genes, page 304, *p* = 0.00012).
Development of sensory organs (29 genes, page 306, *p* = 0.000164).

Only one gene was identified with palatal gene expression, namely TRPS1 (page 358, *p* = 0.00701). Mutations of TRPS1 cause trichorhinopharyngeal (TRPS1) syndrome, which includes a rounded bulbous nose and a long flat upper lip and dental anomalies. Some patients also have heart, kidney, and genitourinary system anomalies.

Only a single functional annotation was identified relevant to nasal development, which involved three genes: BMP4, CHD7, and GLI3 (3 genes, page 343, *p* = 0.00108). Bone morphogenetic protein 4 (BMP4) is the natural antagonist to sonic hedgehog (shh), and both are involved in eye and ear formation. GLI3 (Gli family zinc finger 3) is part of the hedgehog signaling pathway. CHD7 (chromodomain helicase DNA binding protein 7) is a DNA-binding protein.

Many functional annotations were involved in eye formation, including eye formation (25 genes, page 302; 15 genes, page 328), eye morphology (18 genes, page 308; 11 genes page 324; 5 genes, page 335; 9 genes, page 340; 4 genes, page 341), and abnormalities of photoreceptors (5 genes, page 340).

The abnormal morphology of the anterior eye segment was noted (7 genes, page 318). Abnormalities of the corneal stroma were also noted (3 genes, page 323). The morphology of the lens was noted (3 genes, page 354), as was lens formation (4 genes, page 333).

Abnormalities of ear (7 genes, page 342) and auditory (3 genes, page 318) development were noted, as were disruptions to the hair cell morphology (5 genes page 316), inner ear (8 genes, page 316; 9 genes, page 323), cochlea (6 genes, page 324; 4 genes page 353), cochlea duct (2 genes, page 350), spiral ganglion (4 genes, page 312; 2 genes page 342), and vestibulocochlear nerve (3 genes, page 344).

### 4.7. Generalizability

Since this study uses a large European dataset, one of the largest in the world, and has produced results closely in line with those found elsewhere, we feel that the results produced in the present study are robust to external generalization. Moreover, in the present work, we have been careful to adopt a framework of formal quantitative causal inference, meaning that this study moves beyond a simply observational project and becomes a pseudo-randomized analytical environment from which one can appropriately draw causal inferences. It is also noted that there are robust epigenomic aetiopathological biological explanations for the present findings, which also support the epidemiological results. In that the effects described then are causal rather than merely associational, this causal dimension also reinforces the view that these results are widely generalizable.

### 4.8. Strengths and Limitations

This study has a number of strengths and limitations. Its strengths include the use of one of the world’s largest datasets for congenital anomalies together with the drug exposure dataset from the EMCDDA, which is unusually fulsome. We have also been careful to use inverse probability weighting for all the panel regression modelling, which makes our analyses pseudo-randomized and suitable for drawing formal causal inferences from. We have also used E-values liberally throughout this report, which further robustifies the results to external confounding. We have used multiple paneled graphs and plots to present complex time series data at a single glance, which is visually useful to validate the quantitative analyses presented. Ranger regression was used for the formal variable selection. The study’s limitations include the fact that we did not have access to individual participant-level data, which is a relatively common limitation broadly applicable to many epidemiological investigations. In addition, some of the data, such as the daily cannabis use data, was incomplete and had to be interpolated. It is, therefore, important to bear this in mind when considering the results that mention daily use. Data on anomaly rates by sex were not available. This would be a useful refinement for future studies.

## 5. Conclusions

In conclusion, the present study describes how it was possible to relate eight of the nine orofacial congenital anomalies investigated herein to the metrics of cannabis exposure in either a bivariate or inverse probability weighted panel regression and geospatial modelling framework, with high levels of statistical significance and moderate to high levels of minimum E-values signifying robustness in the sensitivity analyses. As well as the usual associational regression techniques, the study uses the techniques for formal quantitative causal inference, which moves the analysis from the world of an observational study into a truly pseudo-randomized casual inferential paradigm in which casual inferences may be properly made. The study results are externally verified by other recent series, with the addition of congenital glaucoma and congenital cataract, which are two new anomalies not previously linked with cannabis exposure. While the implications of the study are concerning in terms of orofacial congenital anomalies themselves, the data are particularly concerning with regard to their implications for fetal brain development and intellectual disability, which is so often paired with anomalies of the anterior head. It is noteworthy that recent epigenomic studies of cannabis dependence and withdrawal appear to provide powerful potential and novel mechanistic explanations for these findings. The intersection of the rapidly rising community cannabis exposure with the known exponential cannabinoid genotoxic dose-response relationship is of particular concern for custodianship by this generation of the genome and epigenome of the generations to come.

## Figures and Tables

**Table 1:** Significant Positive Bivariate Regression Slopes.

Anomaly	Substance	Mean Anomaly Rate	Estimate	Std. Error	Sigma	t_Statistic	*p*_Value	E-Value Estimate	E-Value Lower Bound
Choanal Atresia	Daily.Interpol.	0.8651	16.4363	6.1304	0.5754	2.6811	0.0084	3.89 × 10 ^11^	2.26 × 10^3^
Choanal Atresia	Herb	0.8651	4.6769	1.4339	0.5575	3.2618	0.0014	4.13 × 10^3^	41.97
Choanal Atresia	LMCannabis_Herb	0.8651	5.6312	1.9603	0.5627	2.8727	0.0048	1.80 × 10^4^	36.06
Choanal Atresia	LM.Cannabis_×_Herb.THC_×_Daily.Interpol.	0.8651	2.0059	0.6690	0.5712	2.9983	0.0033	48.36	5.53
Choanal Atresia	Cocaine	0.8651	0.2113	0.0698	0.5607	3.0258	0.0030	2.17	1.51
Choanal Atresia	Daily.Interpol.	0.8651	0.6931	0.3224	0.6033	2.1496	0.0340	5.13	1.43
Cleft lip +/− palate	Herb	8.6394	4.8310	1.4738	0.5731	3.2779	0.0014	4.29 × 10^3^	43.60
Cleft lip +/− palate	Resin	8.6394	1.9931	0.6734	0.6054	2.9596	0.0038	39.50	4.97
Cleft palate	Herb	5.4067	4.5693	1.9872	0.6581	2.2993	0.0235	1.11 × 10^3^	4.58
Cleft lip +/− palate	Amphetamine	8.6394	0.3116	0.0694	0.5535	4.4886	1.66 × 10^−5^	2.73	2.00
Cleft lip +/− palate	Cocaine	8.6394	0.1619	0.0730	0.5863	2.2183	0.0284	1.89	1.21
Cong. Cataract	Daily.Interpol.	1.2577	28.5308	7.0169	0.6586	4.0660	8.88 × 10^−5^	2.63 × 10^17^	1.53 × 10^9^
Cong. Cataract	LM.Cannabis_×_Herb.THC_×_Daily.Interpol.	1.2577	3.0504	0.7745	0.6612	3.9383	1.42 × 10^−4^	132.60	16.03
Cong. Cataract	LMCannabis_Herb	1.2577	5.8011	2.3691	0.6800	2.4487	0.0158	4.70 × 10^3^	9.00
Cong. Cataract	LM.Cannabis_×_Resin.THC_×_Daily.Interpol.	1.2577	1.4541	0.3289	0.6155	4.4210	2.54 × 10^−5^	16.65	6.09
Cong. Cataract	LMCannabis_Resin	1.2577	1.6027	0.5343	0.6398	2.9994	0.0034	19.03	3.84
Cong. Cataract	LM_Cannabis	1.2577	5.9992	2.8266	0.6841	2.1224	0.0359	5.84 × 10^3^	3.14
Cong. Cataract	Cocaine	1.2577	0.2832	0.0828	0.6652	3.4197	8.57 × 10^−4^	2.31	1.64
Cong. Glaucoma	Daily.Interpol.	0.2807	14.6074	3.1705	0.2976	4.6073	1.08 × 10^−5^	5.01 × 10^19^	2.91 × 10^11^
Cong. Glaucoma	LM_Cannabis	0.2807	5.1764	1.2223	0.2958	4.2350	4.50 × 10^−5^	1.65 × 10^7^	1.05 × 10^4^
Cong. Glaucoma	LMCannabis_Herb	0.2807	4.6092	1.0216	0.2933	4.5116	1.51 × 10^−5^	3.25 × 10^6^	6.60 × 10^3^
Cong. Glaucoma	LM.Cannabis_×_Herb.THC_×_Daily.Interpol.	0.2807	1.8797	0.3363	0.2871	5.5899	1.60 × 10^−7^	773.45	95.71
Cong. Glaucoma	LM.Cannabis_×_Resin.THC_×_Daily.Interpol.	0.2807	0.7827	0.1480	0.2769	5.2888	7.49 × 10^−7^	25.67	9.59
Cong. Glaucoma	LMCannabis_Resin	0.2807	0.9308	0.2390	0.2862	3.8938	1.74 × 10^−4^	38.06	8.19
Cong. Glaucoma	Cocaine	0.2807	0.0987	0.0384	0.3088	2.5683	0.0114	2.01	1.35
Ear, face and neck	Log(Amphetamine)	2.6052	0.2087	0.0951	0.7583	2.1950	0.0301	1.89	1.20
Ear, face and neck	Annual_Alcohol	2.6052	0.0779	0.0378	0.7600	2.0616	0.0414	1.43	1.07
Holoprosencephaly ∼	Daily.Interpol.	1.4692	21.5235	7.3229	0.6874	2.9392	0.0040	4.75 × 10^12^	2.76 × 10^4^
Holoprosencephaly ∼	LMCannabis_Herb	1.4692	7.2896	2.3517	0.6751	3.0996	0.0024	3.70 × 10^4^	74.58
Holoprosencephaly ∼	LM_Cannabis	1.4692	6.4729	2.8379	0.6868	2.2808	0.0243	1.06 × 10^4^	6.24
Holoprosencephaly ∼	LM.Cannabis_×_Herb.THC_×_Daily.Interpol.	1.4692	2.2977	0.8069	0.6889	2.8476	0.0052	41.11	4.61
Holoprosencephaly ∼	LMCannabis_Resin	1.4692	1.7077	0.5689	0.6812	3.0017	0.0034	19.06	3.85
Holoprosencephaly ∼	Herb	1.4692	3.8304	1.7701	0.6883	2.1640	0.0324	316.06	2.64
Holoprosencephaly ∼	LM.Cannabis_×_Resin.THC_×_Daily.Interpol.	1.4692	1.0633	0.3702	0.6927	2.8724	0.0050	7.55	2.50
Holoprosencephaly ∼	Cocaine	1.4692	0.2711	0.0838	0.6728	3.2355	0.0016	2.24	1.58
Holoprosencephaly ∼	Resin	1.4692	1.6126	0.7738	0.6956	2.0839	0.0396	15.97	1.53
Oro-facial clefts	Herb	13.8647	5.5992	1.4086	0.4665	3.9751	1.29 × 10^−4^	1.11 × 10^5^	512.89
Oro-facial clefts	Resin	13.8647	1.5100	0.5861	0.5046	2.5763	0.0115	29.95	3.26
Oro-facial clefts	Log(Amphetamine)	13.8647	0.2437	0.0601	0.4653	4.0522	9.75 × 10^−5^	2.60	1.88

Abbreviations: LM.Cannabis—Last-month cannabis use; Herb.THC—THC concentration of cannabis herb; Resin.THC—THC concentration of cannabis herb; Daily.Interpol.—Daily cannabis use interpolated. Holoprosencephaly ∼ refers to the combination of holoprosencephaly and arhinencephaly.

**Table 2:** Geospatial Regression Models of Orofacial Clefts.

Parameter Values			Model Parameters		
Parameter	Estimate (C.I.)	*p*-Value	Parameter	Value	Significance
*Additive*					
*Rate ∼ Tobacco + Alcohol + Herb + Daily.Interpol. + Resin + LM.Cannabis_×_Herb.THC_x_Daily.Interpol. + Amphetamines + Cocaine + Income*					
Tobacco	0.05 (0.03, 0.07)	7.76 × 10^−6^	rho	−0.7032	2.35 × 10^−11^
Herb	5.38 (3.55, 7.21)	8.86 × 10^−9^	lambda	0.4868	5.85 × 10^−5^
LM.Cannabis_×_Herb.THC_×_Daily.Interpol.	−2.62 (−3.53, −1.71)	1.69 × 10^−8^			
Amphetamines	0.12 (0.04, 0.21)	0.0064			
Cocaine	0.24 (0.11, 0.37)	0.0004			
Income	0 (0, 0)	0.0003			
***Interactive***					
Rate ∼ Tobacco * Herb + Daily.Interpol. + LM.Cannabis + LM.Cannabis_×*_Herb.THC_*×*_Daily.Interpol. + Alcohol + Amphetamines + Cocaine + Income*					
Tobacco	−0.04 (−0.08, 0)	0.0490	rho	−0.5114	0.0048
Herb	−18.6 (−29.11, −8.09)	0.0005	lambda	0.2927	0.0932
Daily.Interpol.	−23.7 (−41.71, −5.69)	0.0100			
LM.Cannabis	−11.7 (−18.6, −4.8)	0.0009			
Amphetamines	0.1 (0.01, 0.2)	0.0367			
Cocaine	0.49 (0.31, 0.68)	1.50 × 10^−7^			
Income	0 (0, 0)	0.0072			
Tobacco: Herb	0.96 (0.55, 1.37)	5.54 × 10^−6^			
***2 Lags***					
Rate ∼ Tobacco * Herb + LM.Cannabis_×*_Herb.THC_*×*_Daily.Interpol. + Daily.Interpol. + LM.Cannabis + Alcohol + Amphetamines + Cocaine + Income*					
Herb	−15.1 (−22.49, −7.71)	6.02 × 10^−5^	rho	−0.7646	<2.2 × 10^−16^
Alcohol	−0.07 (−0.11, −0.03)	0.0012	lambda	0.591	3.29 × 10^−8^
Amphetamines	0.23 (0.13, 0.33)	1.02 × 10^−5^			
Cocaine	−0.19 (−0.33, −0.05)	0.0085			
Income	0 (0, 0)	1.96 × 10^−8^			
Tobacco: Herb	0.71 (0.44, 0.98)	3.16 × 10^−7^			

Abbreviations: See Table 1. *—Interaction

**Table 3:** Geospatial Regression Models of Anotia/Microtia.

Parameter Values			Model Parameters		
Parameter	Estimate (C.I.)	*p*-Value	Parameter	Value	Significance
*Additive*					
*Rate ∼ Tobacco + Alcohol + LM.Cannabis_*×*_Resin.THC + LM.Cannabis_*×*_Herb.THC + LM.Cannabis + Resin + Amphetamines + Cocaine + Income*					
Alcohol	0.04 (0.02, 0.06)	0.0004	rho	−0.4636	0.000525
LM.Cannabis_×_Herb.THC	3.56 (1.28, 5.85)	0.0022	lambda	0.4841	1.85 × 10^−5^
LM.Cannabis	−7.78 (−10.88, −4.69)	8.27 × 10^−7^			
Amphetamines	−0.1 (−0.15, −0.04)	0.0003			
Cocaine	0.12 (0.05, 0.2)	0.0007			
***Interactive***					
*Rate ∼ Tobacco + LM.Cannabis_*×_Herb.THC * LM.Cannabis_×*_Resin.THC + Resin + LM.Cannabis + Alcohol + Amphetamines + Cocaine + Income*					
LM.Cannabis_×_Herb.THC	3.56 (1.28, 5.85)	0.0022	rho	−0.4636	0.000527
LM.Cannabis	−7.78 (−10.88, −4.69)	8.27 × 10^−7^	lambda	0.4841	1.84 × 10^−5^
Alcohol	0.04 (0.02, 0.06)	0.0004			
Amphetamines	−0.1 (−0.15, −0.04)	0.0003			
Cocaine	0.12 (0.05, 0.2)	0.0007			
***2 Lags***					
*Rate ∼ Tobacco + LM.Cannabis_*×_Herb.THC * LM.Cannabis_×*_Resin.THC + Resin + LM.Cannabis + Alcohol + Amphetamines + Cocaine + Income*					
LM.Cannabis_×_Herb.THC	7.62 (3.04, 12.2)	0.0011	rho	0.04947	0.908
Resin	−0.79 (−1.58, 0)	0.0500	lambda	0.03795	0.926
LM.Cannabis	−8.16 (−13.17, −3.16)	0.0014			

Abbreviations: See Table 1. In geospatial models, the rho is the spatial coefficient and the lambda is the spatial autocorrelation coefficient. *—Interaction.

**Table 4:** Geospatial Regression Models of Congenital Cataract.

Parameter Values			Model Parameters		
Parameter	Estimate (C.I.)	*p*-Value	Parameter	Value	Significance
*Additive*					
Rate ∼ Tobacco + Alcohol + LM.Cannabis_×_Resin.THC_×_Daily.Interpol. + LM.Cannabis_×_Resin.THC + LM.Cannabis_×_Herb.THC_×_Daily.Interpol. + Herb + Amphetamines + Cocaine + Income					
LM.Cannabis_×_Resin.THC	1.73 (0.69, 2.78)	0.0011	rho	0.3611	0.0161
Amphetamines	−0.29 (−0.42, −0.15)	2.71 × 10^−5^	lambda	−0.3298	0.0134
Cocaine	0.41 (0.27, 0.56)	3.36 × 10^−8^			
***Interactive***					
Rate ∼ Tobacco * LM.Cannabis_×*_Resin.THC_*×*_Daily.Interpol. + Daily.Interpol. + LM.Cannabis_*×*_Herb.THC_*×*_Daily.Interpol. + Herb + Alcohol + Amphetamines + Cocaine + Income*					
LM.Cannabis_×_Resin.THC_×_Daily.Interpol.	6.48 (2.52, 10.43)	0.0013	rho	0.2149	0.191
Daily.Interpol.	74.98 (45.28, 104.67)	7.45 × 10^−7^	lambda	−0.2066	0.128
LM.Cannabis_×_Herb.THC_×_Daily.Interpol.	−8.94 (−14.46, −3.43)	0.0015			
Resin	7.04 (3.3, 10.78)	0.0002			
Amphetamines	−0.31 (−0.45, −0.18)	5.58 × 10^−6^			
Tobacco: LM.Cannabis_×_Resin.THC_×_Daily.Interpol.	−0.16 (−0.32, −0.01)	0.0402			
***2 Lags***					
Rate ∼ Tobacco * LM.Cannabis_×*_Resin.THC_*×*_Daily.Interpol. + Daily.Interpol. + LM.Cannabis_*×*_Herb.THC_*×*_Daily.Interpol. + Herb + Alcohol + Amphetamines + Cocaine + Income*					
LM.Cannabis_×_Resin.THC_×_Daily.Interpol.	8.71 (1.79, 15.62)	0.0136	rho	0.2439	0.107
Daily.Interpol.	35.6 (17.14, 54.06)	0.0002	lambda	−0.2644	0.0364
Amphetamines	−0.32 (−0.47, −0.16)	4.43 × 10^−5^			
Tobacco: LM.Cannabis_×_Resin.THC_×_Daily.Interpol.	−0.3 (−0.56, −0.04)	0.0220			

Abbreviations: See Table 1 and Table 3.

**Table 5:** Geospatial Regression Models of Holoprosencephaly.

Parameter Values			Model Parameters		
Parameter	Estimate (C.I.)	*p*-Value	Parameter	Value	Significance
***Additive***					
*Rate ∼ Tobacco + Alcohol + LM.Cannabis_*×*_Resin.THC_*×*_Daily.Interpol. + LM.Cannabis_*×*_Herb.THC_*×*_Daily.Interpol. + LM.Cannabis_*×*_Resin.THC + Daily.Interpol. + Amphetamines + Cocaine + Income*					
Tobacco	0.05 (0.01, 0.09)	0.0124	rho	0.4943	3.60 × 10^−5^
Alcohol	0.09 (0.02, 0.16)	0.0150	lambda	−0.5753	5.12 × 10^−7^
LM.Cannabis_×_Resin.THC_×_Daily.Interpol.	0.93 (0.36, 1.5)	0.0015			
Amphetamines	−0.23 (−0.37, −0.08)	0.0020			
Income	0 (0, 0)	5.47 × 10^−9^			
***Interactive***					
*Rate ∼ Tobacco + LM.Cannabis_*×*_Resin.THC_*×_Daily.Interpol. * LM.Cannabis_×*_Herb.THC_*×*_Daily.Interpol. + LM.Cannabis_*×*_Resin.THC + Daily.Interpol. + Alcohol + Amphetamines + Cocaine + Income*					
Tobacco	0.05 (0.01, 0.09)	0.0124	rho	0.4943	3.56 × 10^−5^
LM.Cannabis_×_Resin.THC_×_Daily.Interpol.	0.93 (0.36, 1.5)	0.0015	lambda	−0.5753	5.04 × 10^−7^
Alcohol	0.09 (0.02, 0.16)	0.0150			
Amphetamines	−0.23 (−0.37, −0.08)	0.0020			
Income	0 (0, 0)	5.47 × 10^−9^			
***2 Lags***					
*Rate ∼ Tobacco + LM.Cannabis_*×*_Herb.THC_*×_Daily.Interpol. * LM.Cannabis_×*_Resin.THC_*×*_Daily.Interpol. + LM.Cannabis_*×*_Resin.THC + Daily.Interpol. + Alcohol + Amphetamines + Cocaine + Income*					
Tobacco	0.07 (0.03, 0.12)	0.0004	rho	0.3052	0.202
LM.Cannabis_×_Resin.THC_×_Daily.Interpol.	1.14 (0.08, 2.2)	0.0347	lambda	−0.4246	0.0652
Amphetamines	−0.2 (−0.39, −0.02)	0.0314			
Income	0 (0, 0)	8.07 × 10−^6^			

Abbreviations: See Table 1 and Table 3. *—Interaction.

**Table 6:** E-Values from Panel Models.

Anomaly	Term	*p*-Value	E-Value Estimate	Lower Bound E-Value
Anotia	***Additive***			
	LM.Cannabis_×_Herb.THC	1.04 × 10^−8^	1.37 × 10^13^	1.32 × 10^10^
	***Interactive***			
	LM.Cannabis_×_Herb.THC	2.99 × 10^−8^	6.35 × 10^12^	4.88 × 10^8^
	***1 Lag***			
	LM.Cannabis_×_Herb.THC	0.0093	381.64	7.38
	***2 Lags***			
	Tobacco: LM.Cannabis_×_Resin.THC	0.0004	4.96	2.59
Orofacial Clefts	***Additive***			
	Herb	0.0016	398.03	15.73
	***Interactive***			
	Daily.Interpol.	0.0416	1.37 × 10^7^	3.85
	***2 Lags***			
	Tobacco: Herb	2.65 × 10^−5^	4.75	2.85
Congenital Cataract	***Additive***			
	LM.Cannabis_×_Herb.THC: LM.Cannabis_×_Resin.THC_×_Daily.Interpol.	0.0103	13.94	2.66
	***Interactive***			
	LM.Cannabis_×_Resin.THC_×_Daily.Interpol.	5.88 × 10^−16^	19.14	11.68
	***2 Lags***			
	LM.Cannabis_×_Resin.THC_×_Daily.Interpol.	0.0001	1.36 × 10^3^	58.12
Holoprosencephaly	***Additive***			
	LM.Cannabis_×_Resin.THC	3.21 × 10^−13^	11.48	7.29
	***Interactive***			
	LM.Cannabis_×_Herb.THC_×_Daily.Interpol.: Herb	0.0037	7.72	2.61
	***2 Lags***			
	LM.Cannabis_×_Resin.THC	3.67 × 10^−10^	12.29	7.10

Abbreviations: See Table 1.

**Table 7:** E-Values from Geospatial Models.

Anomaly	Term	*p*-Value	E-Value Estimate	Lower Bound E-Value
Anotia	***Additive***			
	LM.Cannabis_×_Herb.THC	0.0022	2.35 × 10^7^	716.97
	***Interactive***			
	LM.Cannabis_×_Herb.THC	0.0022	2.35 × 10^7^	716.97
	***2 Lags***			
	LM.Cannabis_×_Herb.THC	0.0011	1.05 × 10^13^	2.47 × 10^5^
Orofacial Clefts	***Additive***			
	Herb	8.86 × 10^−9^	1.92 × 10^9^	1.69 × 10^6^
	***Interactive***			
	Tobacco: Herb	5.54 × 10^−6^	24.52	7.90
	***2 Lags***			
	Tobacco: Herb	3.16 × 10^−7^	10.59	5.21
Congenital Cataract	***Additive***			
	LM.Cannabis_×_Resin.THC	0.0011	36.32	5.84
	***Interactive***			
	LM.Cannabis_×_Resin.THC_×_Daily.Interpol.	0.0013	9.05 × 10^4^	131.90
	Daily.Interpol.	7.45 × 10−^7^	1.63 × 10^54^	8.02 × 10^32^
	Resin	0.0002	2.31 × 10^5^	479.00
	***2 Lags***			
	LM.Cannabis_×_Resin.THC_×_Daily.Interpol.	0.0136	6.41 × 10^6^	44.01
	Daily.Interpol.	0.0002	8.08 × 10^26^	1.38 × 10^13^
Holoprosencephaly	***Additive***			
	LM.Cannabis_×_Resin.THC_×_Daily.Interpol.	0.0015	9.34	3.10
	***Interactive***			
	LM.Cannabis_×_Resin.THC_×_Daily.Interpol.	0.0015	9.34	3.10
	***2 Lags***			
	LM.Cannabis_×_Resin.THC_×_Daily.Interpol.	0.0347	11.65	1.54

Abbreviations: See Table 1.

**Table 8:** Combined E-Value List from Panel and Spatial Models.

No.	Congenital Anomaly	Regression	Model Type	Term	*p*-Value	E-Value Estimate	Lower Bound E-Value
1	Congenital Cataract	Panel	Interactive	Daily.Interpol.	7.45 × 10^−7^	1.63 × 10^54^	8.02 × 10^32^
2	Congenital Cataract	Panel	2 Lags	Daily.Interpol.	0.0002	8.08 × 10^26^	1.38 × 10^13^
3	Anotia	Spatial	Additive	LM.Cannabis_×_Herb.THC	1.04 × 10^−8^	1.37 × 10^13^	1.32 × 10^10^
4	Anotia	Spatial	Interactive	LM.Cannabis_×_Herb.THC	2.99 × 10^−8^	6.35 × 10^12^	4.88 × 10^8^
5	Orofacial Clefts	Panel	Additive	Herb	8.86 × 10^−9^	1.92 × 10^9^	1.69 × 10^6^
6	Anotia	Panel	2 Lags	LM.Cannabis_×_Herb.THC	0.0011	1.05 × 10^13^	2.47 × 10^5^
7	Anotia	Panel	Additive	LM.Cannabis_×_Herb.THC	0.0022	2.35 × 10^7^	716.97
8	Anotia	Panel	Interactive	LM.Cannabis_×_Herb.THC	0.0022	2.35 × 10^7^	716.97
9	Congenital Cataract	Panel	Interactive	Resin	0.0002	2.31 × 10^5^	479.00
10	Congenital Cataract	Panel	Interactive	LM.Cannabis_×_Resin.THC_×_Daily.Interpol.	0.0013	9.05 × 10^4^	131.90
11	Congenital Cataract	Spatial	2 Lags	LM.Cannabis_×_Resin.THC_×_Daily.Interpol.	0.0001	1.36 × 10^3^	58.12
12	Congenital Cataract	Panel	2 Lags	LM.Cannabis_×_Resin.THC_×_Daily.Interpol.	0.0136	6.41 × 10^6^	44.01
13	Orofacial Clefts	Spatial	Additive	Herb	0.0016	398.03	15.73
14	Congenital Cataract	Spatial	Interactive	LM.Cannabis_×_Resin.THC_×_Daily.Interpol.	5.88 × 10^−16^	19.14	11.68
15	Orofacial Clefts	Panel	Interactive	Tobacco: Herb	5.54 × 10^−6^	24.52	7.90
16	Anotia	Spatial	1 Lag	LM.Cannabis_×_Herb.THC	0.0093	381.64	7.38
17	Holoprosencephaly	Spatial	Additive	LM.Cannabis_×_Resin.THC	3.21 × 10^−13^	11.48	7.29
18	Holoprosencephaly	Spatial	2 Lags	LM.Cannabis_×_Resin.THC	3.67 × 10^−10^	12.29	7.10
19	Congenital Cataract	Panel	Additive	LM.Cannabis_×_Resin.THC	0.0011	36.32	5.84
20	Orofacial Clefts	Panel	2 Lags	Tobacco: Herb	3.16 × 10^−7^	10.59	5.21
21	Orofacial Clefts	Spatial	Interactive	Daily.Interpol.	0.0416	1.37 × 10^7^	3.85
22	Holoprosencephaly	Panel	Additive	LM.Cannabis_×_Resin.THC_×_Daily.Interpol.	0.0015	9.34	3.10
23	Holoprosencephaly	Panel	Interactive	LM.Cannabis_×_Resin.THC_×_Daily.Interpol.	0.0015	9.34	3.10
24	Orofacial Clefts	Spatial	2 Lags	Tobacco: Herb	2.65 × 10^−5^	4.75	2.85
25	Congenital Cataract	Spatial	Additive	LM.Cannabis_×_Herb.THC: LM.Cannabis_×_Resin.THC_×_Daily.Interpol.	0.0103	13.94	2.66
26	Holoprosencephaly	Spatial	Interactive	LM.Cannabis_×_Herb.THC_×_Daily.Interpol.: Herb	0.0037	7.72	2.61
27	Anotia	Spatial	2 Lags	Tobacco: LM.Cannabis_×_Resin.THC	0.0004	4.96	2.59
28	Holoprosencephaly	Panel	2 Lags	LM.Cannabis_×_Resin.THC_×_Daily.Interpol.	0.0347	11.65	1.54

Abbreviations: See Table 1.

**Table 9:** Ordered List of E-Values.

No.	E-Value Estimate	Lower Bound E-Value
1	1.63 × 10^54^	8.02 × 10^32^
2	8.08 × 10^26^	1.38 × 10^13^
3	1.37 × 10^13^	1.32 × 10^10^
4	1.05 × 10^13^	4.88 × 10^8^
5	6.35 × 10^12^	1.69 × 10^6^
6	1.92 × 10^9^	2.47 × 10^5^
7	2.35 × 10^7^	716.97
8	2.35 × 10^7^	716.97
9	1.37 × 10^7^	479.00
10	6.41 × 10^6^	131.90
11	2.31 × 10^5^	58.12
12	9.05 × 10^4^	44.01
13	1.36 × 10^3^	15.73
14	398.03	11.68
15	381.64	7.90
16	36.32	7.38
17	24.52	7.29
18	19.14	7.10
19	13.94	5.84
20	12.29	5.21
21	11.65	3.85
22	11.48	3.10
23	10.59	3.10
24	9.34	2.85
25	9.34	2.66
26	7.72	2.61
27	4.96	2.59
28	4.75	1.54

Table key: Note that both lists of E-value estimates and lower bounds are presented in descending order. This implies that the paired relationship between the values has been broken.

**Table 10:** E-Values Listed by Orofacial Congenital Anomaly.

No.	Congenital Anomaly	Regression	Model Type	Term	*p*-Value	E-Value Estimate	Lower Bound E-Value
1	Anotia	Spatial	Additive	LM.Cannabis_x_Herb.THC	1.04 × 10^−8^	1.37 × 10^13^	1.32 × 10^10^
2	Anotia	Spatial	Interactive	LM.Cannabis_x_Herb.THC	2.99 × 10^−8^	6.35 × 10^12^	4.88 × 10^8^
3	Anotia	Panel	2 Lags	LM.Cannabis_x_Herb.THC	0.0011	1.05 × 10^13^	2.47 × 10^5^
4	Anotia	Panel	Additive	LM.Cannabis_x_Herb.THC	0.0022	2.35 × 10^7^	716.97
5	Anotia	Panel	Interactive	LM.Cannabis_x_Herb.THC	0.0022	2.35 × 10^7^	716.97
6	Anotia	Spatial	1 Lag	LM.Cannabis_x_Herb.THC	0.0093	381.64	7.38
7	Anotia	Spatial	2 Lags	Tobacco: LM.Cannabis_x_Resin.THC	0.0004	4.96	2.59
8	Congenital Cataract	Panel	Interactive	Daily.Interpol.	7.45 × 10^−7^	1.63 × 10^54^	8.02 × 10^32^
9	Congenital Cataract	Panel	2 Lags	Daily.Interpol.	0.0002	8.08 × 10^26^	1.38 × 10^13^
10	Congenital Cataract	Panel	Interactive	Resin	0.0002	2.31 × 10^5^	479.00
11	Congenital Cataract	Panel	Interactive	LM.Cannabis_x_Resin.THC_x_Daily.Interpol.	0.0013	9.05 × 10^4^	131.90
12	Congenital Cataract	Spatial	2 Lags	LM.Cannabis_x_Resin.THC_x_Daily.Interpol.	0.0001	1.36 × 10^3^	58.12
13	Congenital Cataract	Panel	2 Lags	LM.Cannabis_x_Resin.THC_x_Daily.Interpol.	0.0136	6.41 × 10^6^	44.01
14	Congenital Cataract	Spatial	Interactive	LM.Cannabis_x_Resin.THC_x_Daily.Interpol.	5.88 × 10^−16^	19.14	11.68
15	Congenital Cataract	Panel	Additive	LM.Cannabis_x_Resin.THC	0.0011	36.32	5.84
16	Congenital Cataract	Spatial	Additive	LM.Cannabis_x_Herb.THC: LM.Cannabis_x_Resin.THC_x_Daily.Interpol.	0.0103	13.94	2.66
17	Holoprosencephaly	Spatial	Additive	LM.Cannabis_x_Resin.THC	3.21 × 10^−13^	11.48	7.29
18	Holoprosencephaly	Spatial	2 Lags	LM.Cannabis_x_Resin.THC	3.67 × 10^−10^	12.29	7.10
19	Holoprosencephaly	Panel	Additive	LM.Cannabis_x_Resin.THC_x_Daily.Interpol.	0.0015	9.34	3.10
20	Holoprosencephaly	Panel	Interactive	LM.Cannabis_x_Resin.THC_x_Daily.Interpol.	0.0015	9.34	3.10
21	Holoprosencephaly	Spatial	Interactive	LM.Cannabis_x_Herb.THC_x_Daily.Interpol.: Herb	0.0037	7.72	2.61
22	Holoprosencephaly	Panel	2 Lags	LM.Cannabis_x_Resin.THC_x_Daily.Interpol.	0.0347	11.65	1.54
23	Orofacial Clefts	Panel	Additive	Herb	8.86 × 10^−9^	1.92 × 10^9^	1.69 × 10^6^
24	Orofacial Clefts	Spatial	Additive	Herb	0.0016	398.03	15.73
25	Orofacial Clefts	Panel	Interactive	Tobacco: Herb	5.54 × 10^−6^	24.52	7.90
26	Orofacial Clefts	Panel	2 Lags	Tobacco: Herb	3.16 × 10^−7^	10.59	5.21
27	Orofacial Clefts	Spatial	Interactive	Daily.Interpol.	0.0416	1.37 × 10^7^	3.85
28	Orofacial Clefts	Spatial	2 Lags	Tobacco: Herb	2.65 × 10^−5^	4.75	2.85

Abbreviations: See Table 1.

**Table 11:** Summary of E-Values by Orofacial Congenital Anomaly.

Anomaly	Number	Mean Minimum E-Value	Median Minimum E-Value	Min Minimum E-Value	Max Minimum E-Value	Mean E-Value Estimate	Median E-Value Estimate	Min E-Value Estimate	Max E-Value Estimate
Anotia	7	1.96 × 10^9^	716.97	2.59	1.32 × 10^10^	4.36 × 10^12^	2.35 × 10^7^	4.96	1.37 × 10^13^
Congenital Cataract	9	8.91 × 10^31^	58.12	2.66	8.02 × 10^32^	1.81 × 10^53^	9.05 × 10^4^	13.94	1.63 × 10^54^
Orofacial Clefts	6	2.82 × 10^5^	6.56	2.85	1.69 × 10^6^	3.22 × 10^8^	211.27	4.75	1.92 × 10^9^
Holoprosencephaly	6	4.12	3.1	1.54	7.29	10.30	10.41	7.72	12.29

**Table 12:** Comparison of E-Values by Cannabis Metric.

Group	Number	Mean Minimum E-Value	Median Minimum E-Value	Minimum Minimum E-Value	Maximum Minimum E-Value	Mean E-Value Estimate	Median E-Value Estimate	Minimum E-Value Estimate	Maximum E-Value Estimate
Daily	3	2.67 × 10^32^	1.38 × 10^13^	3.85	8.02 × 10^32^	5.43 × 10^53^	8.08 × 10^26^	1.37 × 10^7^	1.63 × 10^54^
Herb	13	1.05 × 10^9^	15.73	2.61	1.32 × 10^10^	2.35015 × 10^12^	398.03	4.75	1.37 × 10^13^
Resin	12	62.94	7.195	1.54	479	561,081.21	15.715	4.96	6.41 × 10^6^

## Data Availability

All the data generated or analyzed during in study are included in this published article and its Supplementary Materials files. The data, along with the relevant R code, have been made publicly available on the Mendeley Database Repository and can be accessed from these URLs: https://doi.org/10.17632/tysn37t426.1 and https://doi.org/10.17632/jjhpfxz5m7.1 (accessed on 11 December 2022).

## Supplementary Materials

The following supporting information can be downloaded at https://www.mdpi.com/article/10.3390/jox13010006/s1, Table S1: Overall Study Profile; Table S2: Daily Cannabis Use—Raw Data; Table S3: Daily Cannabis Use—Interpolated Data; Table S4: All regression slopes and results from bivariate analyses; Table S5: Variable Importance Tables from Ranger random forest regression—Anotia; Table S6: Variable Importance Tables from Ranger random forest regression—Orofacial clefts; Table S7: Variable Importance Tables from Ranger random forest regression—Congenital cataracts; Table S8: Variable Importance Tables from Ranger random forest regression – Holoprosencephaly; Table S9: Inverse probability weighted panel regression results – Anotia; Table S10: Inverse probability weighted panel regression results—Orofacial clefts; Table S11: Inverse probability weighted panel regression results—Congenital cataracts; Table S12: Inverse probability weighted panel regression results – Holoprosencephaly; Table S13: Inverse probability weighted panel regression results – Holoprosencephaly; Figure S1: Sequential map-graphs of log (choanal atresia rates) across surveyed European nations over time 2010–2019; Figure S2: Sequential map-graphs of last month cannabis use x cannabis resin THC concentration across surveyed European nations over time 2010-2019; Figure S3: Bivariate Map of Log Choanal Atresia by Cannabis Herb THC Concentration across European natoins over time; Figure S4: Geospatial linked between european nations (A) edited and (B) final.
