# Supplementary material for: Geospatiotemporal and Causal Inferential Study of European Epidemiological Patterns of Cannabis- and Substance-Related Congenital Orofacial Anomalies
Source: J Xenobiot. 2023 Feb 1;13(1):42–74. doi: 10.3390/jox13010006 (PMC9944119; doi:10.3390/jox13010006)
Supplement: Supplementary file 1 [file jox-13-00006-s001.zip › jox-2153582-supplementary.pdf]

SF1

Choanal Atresia

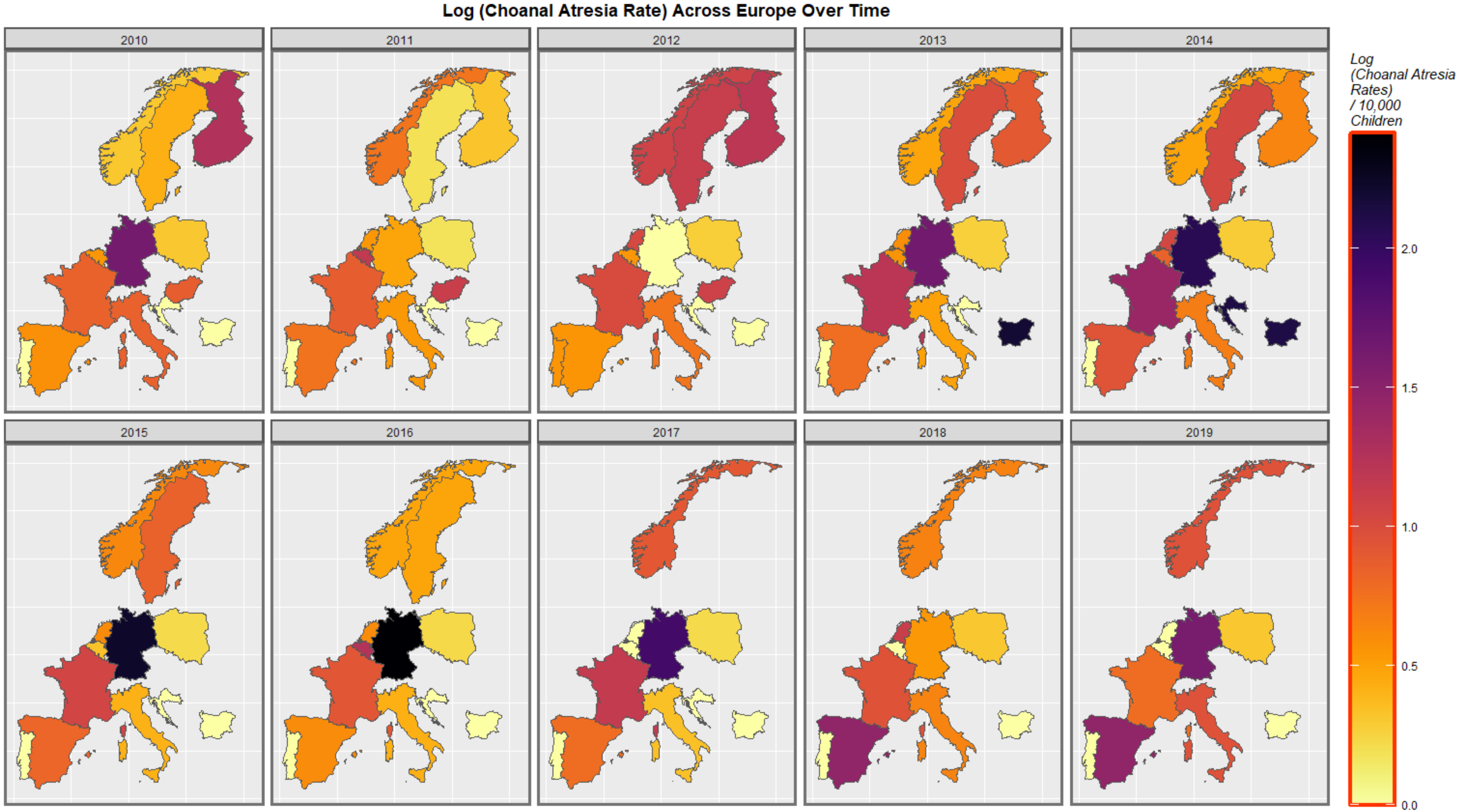

SF2

pmResin

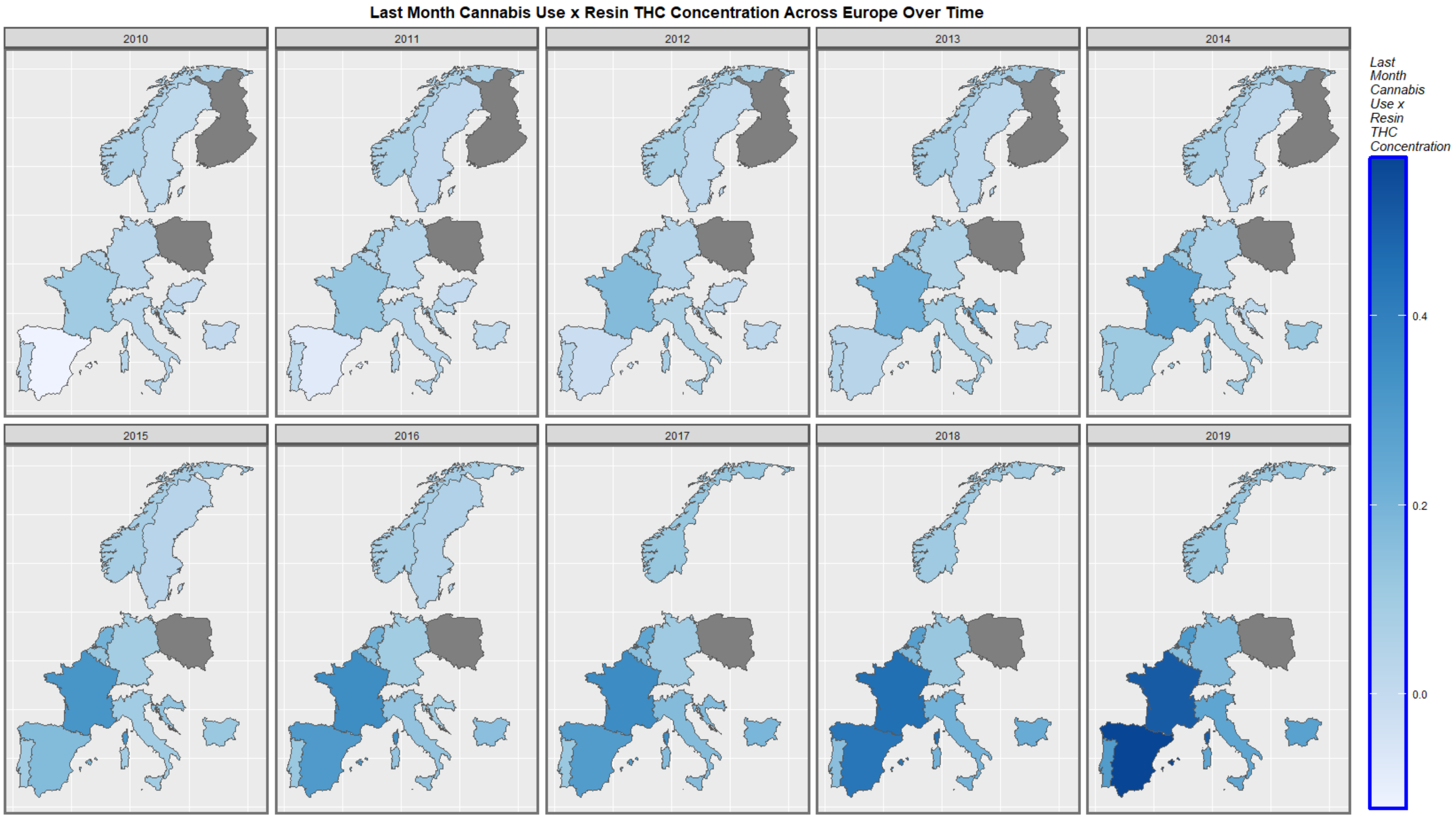

Last Month Cannabis Use x Resin THC Concentration Across Europe Over Time
2010
2011
2012
2013
2014
2015
2016
2017
2018
2019
Last Month Cannabis Use x Resin THC Concentration
0.4
0.2
0.0

SF3 Choanal Atresia ~ Herb Bivariate

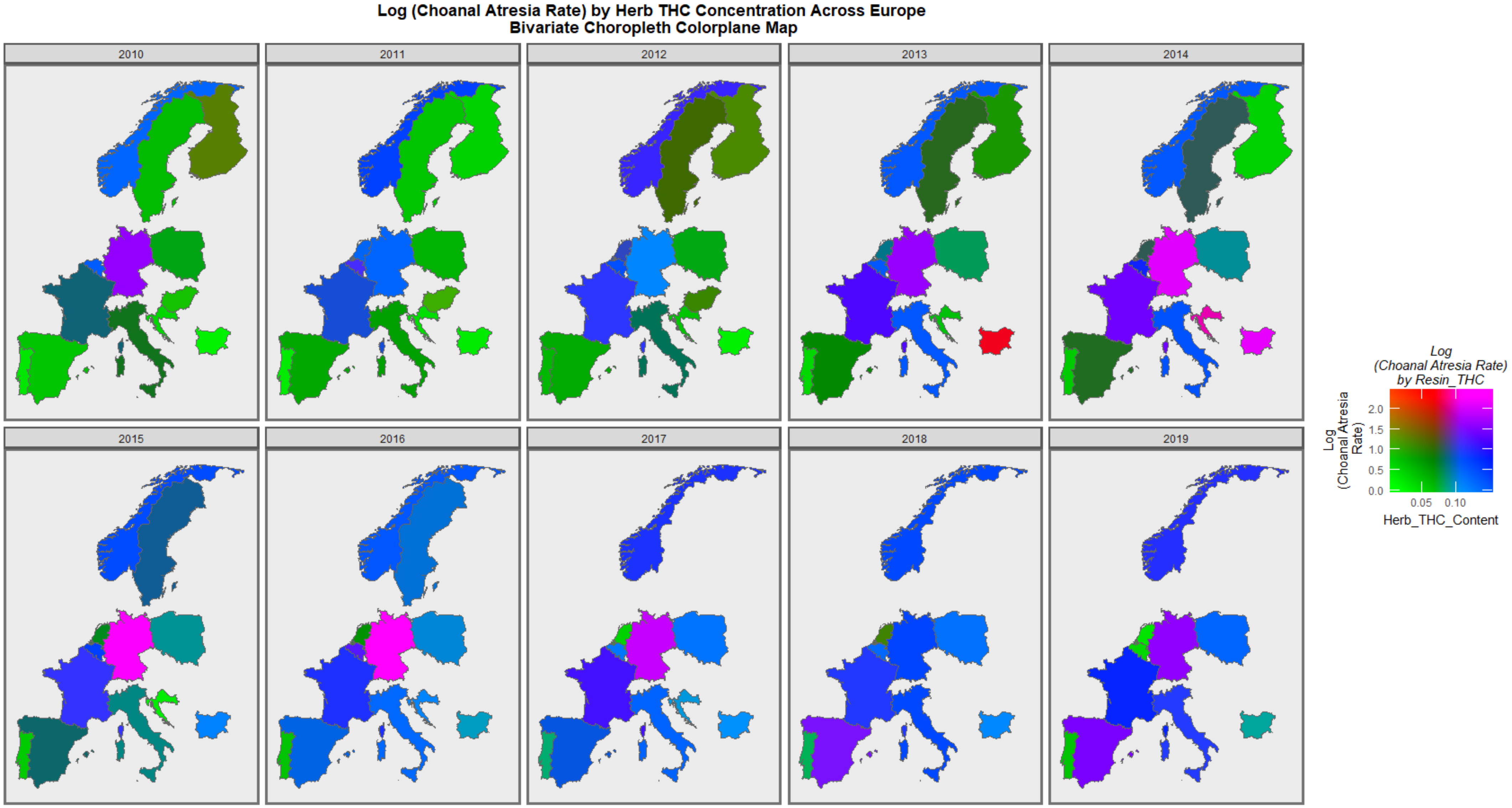

SF4

Edited (Red) Links (Blue) to European Country Neighbourhood Links for General Anomalies Dataset

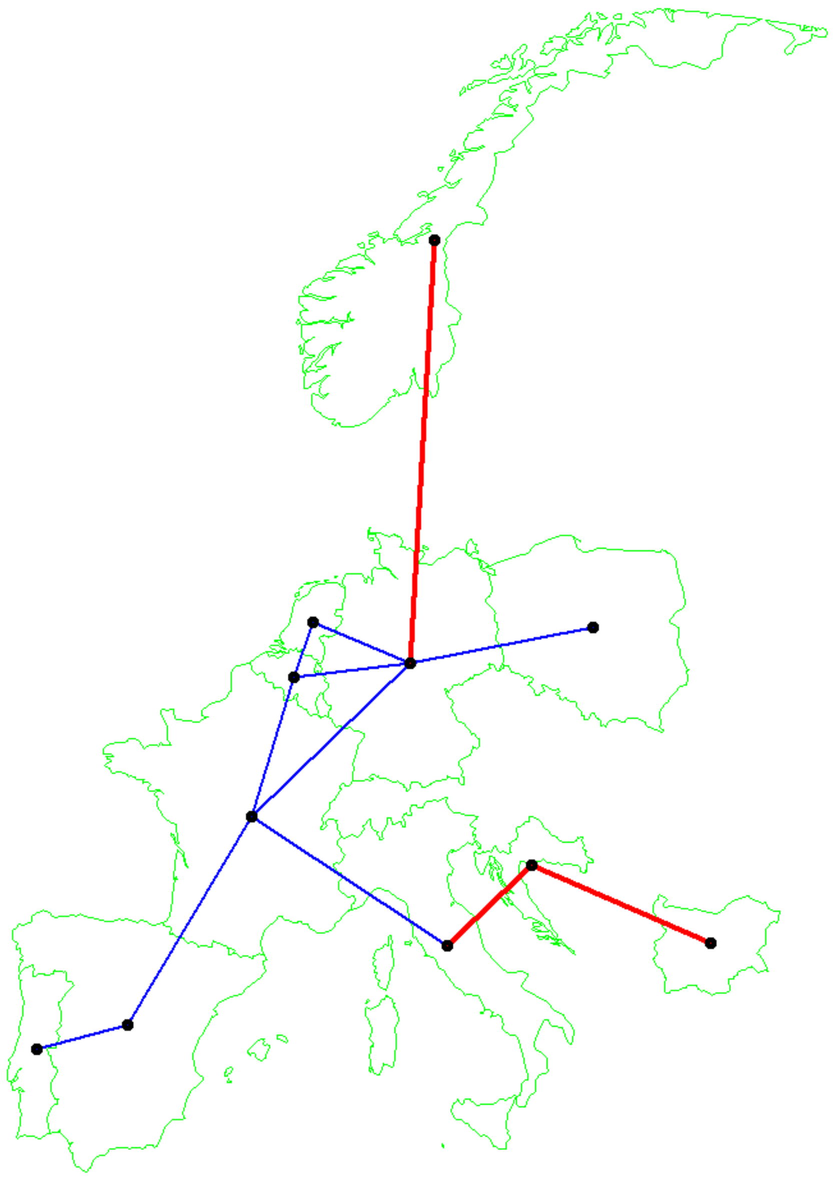

Final Country Neighbourhood Links for European General Anomalies Dataset

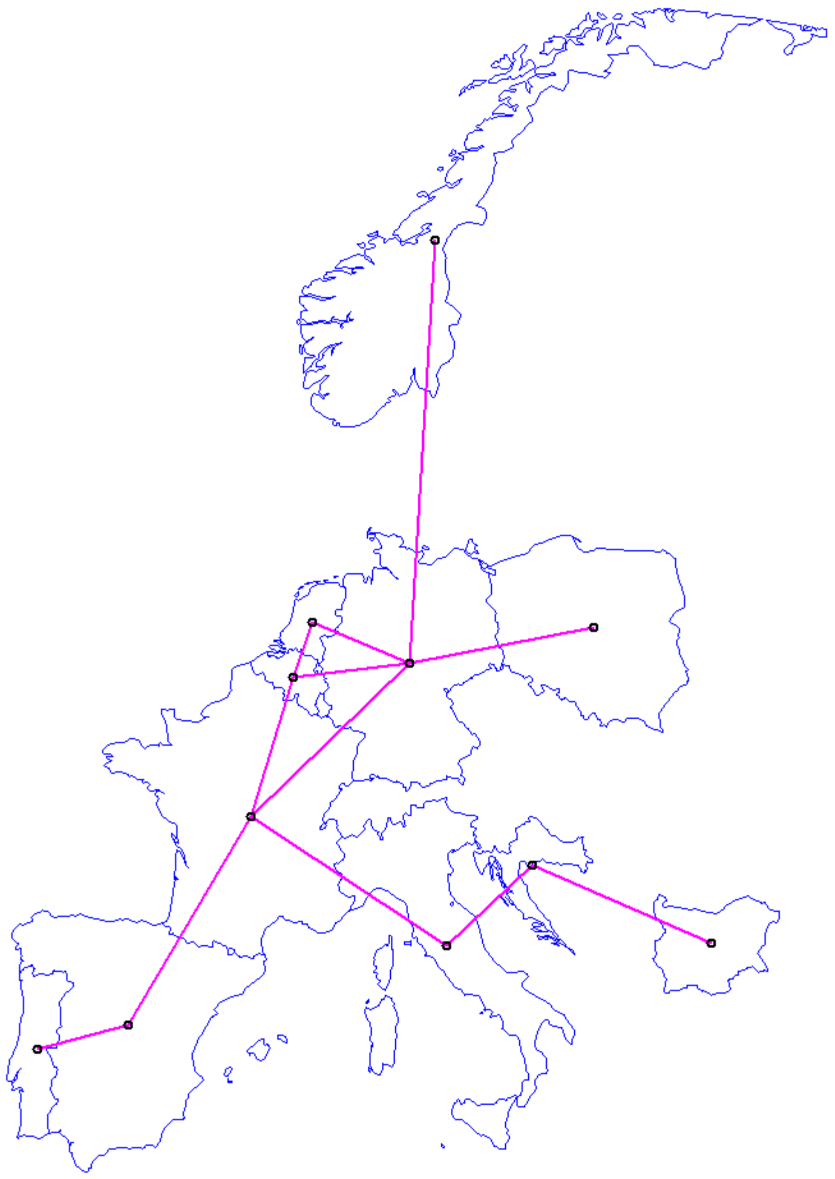

Supplementary Figure Captions

Figure S1: Sequential map-graphs of log (choanal atresia rates) across surveyed European nations over time 2010-2019.

Figure S2: Sequential map-graphs of last month cannabis use x cannabis resin THC concentration across surveyed European nations over time 2010-2019.

Figure S3: Bivariate Map of Log Choanal Atresia by Cannabis Herb THC Concentration across European natoins over time.

Figure S4: Geospatial linked between european nations (A) edited and (B) final.
